# RANKL/RANK is required for cytokine-induced beta cell death; osteoprotegerin, a RANKL inhibitor, reverses rodent type 1 diabetes

**DOI:** 10.1126/sciadv.adf5238

**Published:** 2023-11-01

**Authors:** Nagesha Guthalu Kondegowda, Joanna Filipowska, Jeong-su Do, Nancy Leon-Rivera, Rosemary Li, Rollie Hampton, Selassie Ogyaadu, Camilla Levister, Josef M. Penninger, Helena Reijonen, Carol J. Levy, Rupangi C. Vasavada

**Affiliations:** ^1^ Department of Translational Research and Cellular Therapeutics, Arthur Riggs Diabetes and Metabolism Research Institute, City of Hope, Duarte, CA 91010, USA.; ^2^ Diabetes, Obesity, and Metabolism Institute, Icahn School of Medicine at Mount Sinai, New York, NY 10029, USA.; ^3^ Department of Immunology and Theranostics, Arthur Riggs Diabetes and Metabolism Research Institute, City of Hope, Duarte, CA 91010, USA.; ^4^ Division of Endocrinology and Bone Disease, Icahn School of Medicine at Mount Sinai, New York, NY 10029, USA.; ^5^ IMBA, Institute of Molecular Biotechnology of the Austrian Academy of Sciences, Vienna 1030, Austria.; ^6^ Department of Medical Genetics, Life Sciences Institute, University of British Columbia, Vancouver, Canada.; ^7^ Mindich Child Health and Development Institute, Icahn School of Medicine at Mount Sinai, New York, NY 10029, USA.

## Abstract

Treatment for type 1 diabetes (T1D) requires stimulation of functional β cell regeneration and survival under stress. Previously, we showed that inhibition of the RANKL/RANK [receptor activator of nuclear factor kappa Β (NF-κB) ligand] pathway, by osteoprotegerin and the anti-osteoporotic drug denosumab, induces rodent and human β cell proliferation. We demonstrate that the RANK pathway mediates cytokine-induced rodent and human β cell death through RANK-TRAF6 interaction and induction of NF-κB activation. Osteoprotegerin and denosumab protected β cells against this cytotoxicity. In human immune cells, osteoprotegerin and denosumab reduce proinflammatory cytokines in activated T-cells by inhibiting RANKL-induced activation of monocytes. In vivo, osteoprotegerin reversed recent-onset T1D in nonobese diabetic/Ltj mice, reduced insulitis, improved glucose homeostasis, and increased plasma insulin, β cell proliferation, and mass in these mice. Serum from T1D subjects induced human β cell death and dysfunction, but not α cell death. Osteoprotegerin and denosumab reduced T1D serum–induced β cell cytotoxicity and dysfunction. Inhibiting RANKL/RANK could have therapeutic potential.

## Teaser

OPG and osteoporosis drug denosumab inhibit RANKL/RANK mediated cytokine-induced β cell death and OPG reverts rodent T1D.

## INTRODUCTION

Type 1 diabetes (T1D) is an autoimmune disease in which there is a progressive loss of functional β cells (*1*–*5*). Successful treatment for T1D will involve not only targeting autoimmunity but also enhancing the replication, function, and survival of the residual pancreatic β cells. Receptor activator of nuclear factor kappa Β (NF-κB) (RANK; TNFRSF11A), RANK ligand (RANKL; TNFSF11), osteoprotegerin (OPG; TNFRSF11B), a pathway first described in immune cells and bone, is active in numerous tissues (*6*–*9*), including pancreatic islets and β cells (*10*, *11*). OPG is a secreted decoy receptor that binds RANKL, thereby inhibiting its interaction with its transmembrane receptor, RANK (*6*–*9*). We previously showed that RANKL/RANK is a brake for β cell replication and that recombinant OPG can counteract this brake by binding RANKL to stimulate mouse and human β cell proliferation in vitro, and in vivo, in young, aged, and streptozotocin-treated mice (*12*). OPG increases β cell proliferation in a rat model of intrauterine growth retardation (*13*). Denosumab (DMB), a Food and Drug Administration (FDA)–approved osteoporosis drug, is a human RANKL-specific antibody that acts by inhibiting RANKL/RANK interaction (*14*–*16*). We found that DMB induces human β cell proliferation in vitro and in vivo in human islets transplanted into immunodeficient euglycemic mice (*12*). Thus, inhibition of RANKL/RANK induces rodent and human pancreatic β cell proliferation.

RANKL/RANK activation induces death in various cell types (*17*–*21*). OPG improves survival in endothelial cells; INS-1 cells, a rodent insulinoma cell line; and rat β cells (*11*, *22*, *23*). In this study, we show that OPG and DMB improve primary mouse and human β cell health against stressors relevant to T1D. OPG inhibited cytokine-induced β cell death and reduced proinflammatory NF-κB and signal transducer and activator of transcription 1 (STAT1) signaling in primary mouse and human β cells. OPG also improved β cell function impaired by cytokine treatment in mouse islets. We demonstrated that the RANKL/RANK pathway is required for cytokine-induced rodent and human β cell death using three independent approaches: (i) genetic deletion of the transmembrane receptor RANK in mouse islet cells, (ii) competition between RANKL and OPG proteins, and (iii) use of the RANKL antibody DMB in human islet cells. Furthermore, we showed that interaction of RANK with its intracellular signaling adaptor molecule tumor necrosis factor (TNF) receptor–associated factor 6 (TRAF6) is critical for cytokine-induced cell death in INS1 and primary mouse β cells and is required for nuclear translocation and activation of NF-κB, using two distinct inhibitors, chemical and peptide, to disrupt RANK-TRAF6 interaction.

RANKL/RANK/OPG are expressed in immune cells and play an essential role in immune cell development and function (*8*, *9*, *24*, *25*). We show that in human peripheral blood mononuclear cells (PBMCs), RANK is present on B cells and monocytes, whereas activation of T cells induced intracellular RANKL. Functionally, OPG and DMB suppressed proinflammatory cytokine stimulation in activated T cells, likely through suppressing RANKL-mediated activation of monocytes.

We used the nonobese diabetic (NOD)/Ltj female mouse as a preclinical model of T1D (*26*–*29*) to test the therapeutic potential of OPG. Our in vivo findings show that OPG not only reduces insulitis but also reverses recent-onset T1D in a dose-dependent manner. There is significant improvement in blood glucose, plasma insulin, glucose tolerance, β cell proliferation, and β cell mass, resulting in the reversal of diabetes in OPG-treated versus control saline- or immunoglobulin G (IgG)–treated diabetic NOD mice.

We developed an in vitro culture model to reflect human disease more closely. We find that human islet cells or islets cultured for 24 hours in media in which the fetal calf serum (FCS, 10% v/v) is substituted with serum from patients with T1D show increased human β cell death equivalent to that induced by proinflammatory cytokines and cause β cell dysfunction. In contrast, serum from age- and sex-matched control nondiabetic (ND) subjects does not induce β cell death or β cell dysfunction. The T1D serum cell death assay phenocopies disease pathology in that T1D serum–induced cytotoxicity is specific to β cells and does not induce α cell death. OPG and DMB, but not the IgG control, independently protect human β cells against T1D serum–induced cytotoxicity and improve β cell function, implying that RANKL/RANK is involved in the T1D serum cytotoxic effect. Findings from this study implicate a role for the RANKL/RANK pathway in the pathophysiology of T1D.

## RESULTS

### OPG decreases β cell death, alleviates proinflammatory signaling pathways, and restores β cell function in cytokine-treated mouse islets

**Fig. 1.**
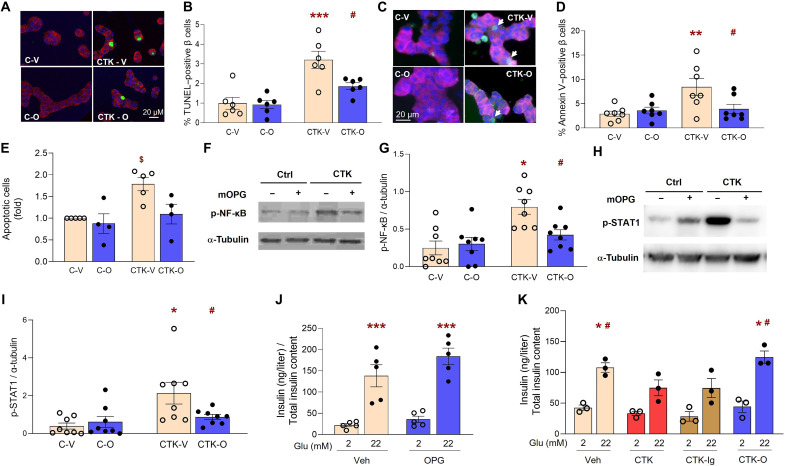
OPG reduces β cell death, proinflammatory signaling pathway activation, and β cell dysfunction induced by cytokines in mouse islets. Mouse islet cells treated without (Ctrl; C) or with cytokines (CTK), and vehicle (Veh; V) or mouse OPG (100 ng/ml; O) for 16 to 24 hours (**A**) were stained for insulin (red), TUNEL (green), and 4′,6-diamidino-2-phenylindole (DAPI) (blue), as represented in the confocal images; and (**B**) quantified for percent TUNEL-positive β cells (*n* = 6); or (**C**) were stained for insulin (red), annexin V (green), and DAPI (blue); white arrows indicate annexin V/insulin double-positive cells and (**D**) quantified for percent annexin V–positive β cells (*n* = 7). White bar indicates the scale for the immunofluorescent images. (**E**) Fold change in annexin V/PI–positive apoptotic INS1 cells assayed by flow cytometry after treatment without (C) or with cytokines (CTK) and vehicle (V) or OPG (1000 ng/ml; O) for 16 hours (*n* = 4 to 5); basal cell death in C-V was 6.88 ± 1.57%. Representative Western blot analysis and quantification of the ratio with tubulin of (**F** and **G**) p-NF-κB and (**H** and **I**) p-STAT1, in mouse islets treated without or with CTK, and Veh or OPG (100 ng/ml) for 24 hours (*n* = 6 to 7). *P < 0.05, **P < 0.01, ***P < 0.001 versus C-V; ^#^*P* < 0.05 versus CTK-V; ^$^*P* < 0.05 versus all other groups. Secreted insulin (nanograms per milliliter)/total insulin content at 2.2 and 22.2 mM glucose from mouse islets (**J**) treated with Veh or OPG (100 ng/ml) for 45 min (*n* = 5); or (**K**) treated with Veh, CTK, CTK + IgG (100 ng/ml), or CTK + OPG (100 ng/ml) for 24 hours (*n* = 3); *P < 0.05, ***P < 0.001 versus 2.2 mM of the same treatment group; ^#^*P* < 0.05 versus 22.2 mM of CTK and CTK-Ig groups. Experiments were done in duplicate or triplicate; 5 to 10 fields and 1545 ± 183 β cells per sample were analyzed for (A) to (D). Individual symbols in the graphs represent independent experiments from individual mice, averaging duplicate (B, D, E, J, and K) or single (G and I) samples. All data represent means ± SEM. All statistical analysis was by analysis of variance (ANOVA) with Tukey’s post hoc analysis.

Proinflammatory cytokines are a key mediator of β cell death and dysfunction in T1D (*30*). We tested whether OPG attenuates cytokine-induced β cell death, dysfunction, and signaling pathway activation in rodent β cells. β cell death was assessed by three different methods, insulin/terminal deoxynucleotidyl transferase–mediated deoxyuridine triphosphate nick end labeling (TUNEL) (Fig. 1, A and B) and insulin/annexin V (Fig. 1, C and D) costaining, in mouse islet cell cultures, and flow cytometry analysis of annexin V/PI (propidium iodide)–stained INS1 cells (Fig. 1E), pretreated with OPG and exposed to proinflammatory mouse cytokines: 50 U of interleukin-1β (IL-1β) (0.091 ng/ml), 1000 U of interferon-γ (IFN-γ) (118.62 ng/ml), and 1000 U of TNF-α (3.7 ng/ml). OPG significantly reduced cytokine-induced mouse β cell death assessed by TUNEL (Fig. 1, A and B) or annexin V (Fig. 1, C and D) staining (1.86 ± 0.17 or 3.9 ± 0.96%) compared to vehicle treatment (3.21 ± 0.44 or 8.4 ± 1.78%), respectively. Similarly, in cytokine-treated INS1 cells, OPG significantly reduced (7.92 ± 1.57%) the percentage of apoptotic annexin V/PI–positive INS1 cells compared to vehicle treatment (11.89 ± 2.57%) (Fig. 1E). Two key signaling pathways activated by cytokines, p-NF-κB-Ser536 (Fig. 1, F and G) and p-Stat1-Ser727 (Fig. 1, H and I), were significantly reduced with OPG treatment in mouse islets, by Western blot analysis. Although OPG did not affect β cell function, as assessed by glucose-stimulated insulin secretion (GSIS), in mouse islets under basal conditions (Fig. 1J), it completely restored the β cell function impaired by cytokine treatment in mouse islets (Fig. 1K). However, hIgG-Fc, used as a control to account for any effects of the Fc fragment in the recombinant OPG-Fc protein, did not improve β cell function in cytokine-treated islets (Fig. 1K). There was no significant change in total insulin content of the islets or insulin stimulation index under these conditions (fig. S1, A to D).

### OPG protects primary human β cells and reduces cytokine-induced activation of proinflammatory signaling pathways

**Fig. 2.**
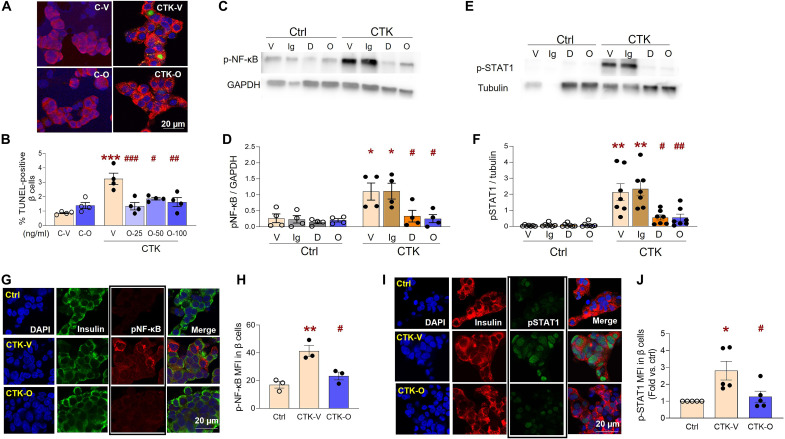
OPG enhances survival, and OPG and DMB reduce activation of proinflammatory signaling pathways induced by cytokines in primary human β cells. Human islet cells treated without (Ctrl; C) or with cytokines (CTK), and vehicle (Veh; V) or human OPG (OPG, O) at 25 to 100 ng/ml (O-25, O-50, O-100) (**A**) were stained for insulin (red), TUNEL (green), and DAPI (blue), as represented in the confocal images; and (**B**) quantified for percent TUNEL-positive β cells after 24 hours (*n* = 4 in duplicate; 5 to 10 fields and 1145 ± 252 β cells per sample were analyzed). Representative Western blot analysis and quantification of the ratio with glyceraldehyde-3-phosphate dehydrogenase (GAPDH) or tubulin of (**C** and **D**) p-NF-κB and (**E** and **F**) p-STAT1, in human islets treated without (Ctrl) or with CTK, and vehicle (V), IgG (100 ng/ml; Ig), DMB (100 ng/ml; D), or OPG (100 ng/ml; O) for 24 hours (*n* = 4 to 7). Human islet cells treated without (Ctrl) or with CTK, and vehicle (V) or OPG (O) (**G**) were stained for p-NF-κB (red), insulin (green), and DAPI (blue), as represented in the individual and merged confocal images; (**H**) quantified for β cell–specific MFI of p-NF-κB after 90 min, (*n* = 3); or (**I**) were stained for p-STAT1 (green), insulin (red), and DAPI (blue), as represented in the individual and merged confocal images and (**J**) quantified for β cell–specific MFI of p-STAT1 after 15 min (*n* = 5), *P < 0.05, **P < 0.01, ***P < 0.001 versus Ctrl or C-V; ^#^*P* < 0.05, ^##^*P* < 0.01, ^###^*P* < 0.001 versus CTK-V and CTK-Ig. White bar indicates the scale for the immunofluorescent images. Individual symbols in the graphs represent independent experiments on different human islet preps, averaging duplicate (B) or single (D, F, H, and J) samples. All data represent means ± SEM. All statistical analysis was by ANOVA with Tukey’s post hoc analysis.

To examine the effect of OPG on human β cell survival, human islet cells were treated with species-specific OPG and cytokines, 50 U of IL-1β (0.714 ng/ml), 1000 U of IFN-γ (50 ng/ml), and 1000 U of TNF-α (13.15 ng/ml), and assayed for β cell death by insulin-TUNEL costaining (Fig. 2A). OPG at 25 to 100 ng/ml significantly reduced β cell death (1.34 ± 0.26% to 1.62 ± 0.31%) compared to cytokine treatment (3.24 ± 0.4%) alone (Fig. 2B). Inflammatory signaling pathways, p-NF-κB-Ser536 (Fig. 2, C and D) and p-Stat1-Ser727 (Fig. 2, E and F), activated by cytokines in human islets, were significantly reduced by OPG, but not IgG, as quantified by Western blot analysis. As ~50% of human islets are composed of non-β cells (*31*), we examined the effect of OPG on cytokine-induced signaling in β cells at the cellular level by costaining human islet cells for p-NF-κB-Ser536 (Fig. 2G) or p-STAT1-Ser727 (Fig. 2I) and insulin. Cytokines increased mean fluorescence intensity (MFI) of p-NF-κB (41.22 ± 4.04%) and p-STAT1 (2.81 ± 0.56%) staining in human β cells, which was significantly reduced to control levels with OPG treatment for p-NF-κB (23.27 ± 2.81%) (Fig. 2H) and p-STAT1 (1.27 ± 0.33%) (Fig. 2J).

### Cytokine-induced rodent β cell death requires RANK

**Fig. 3.**
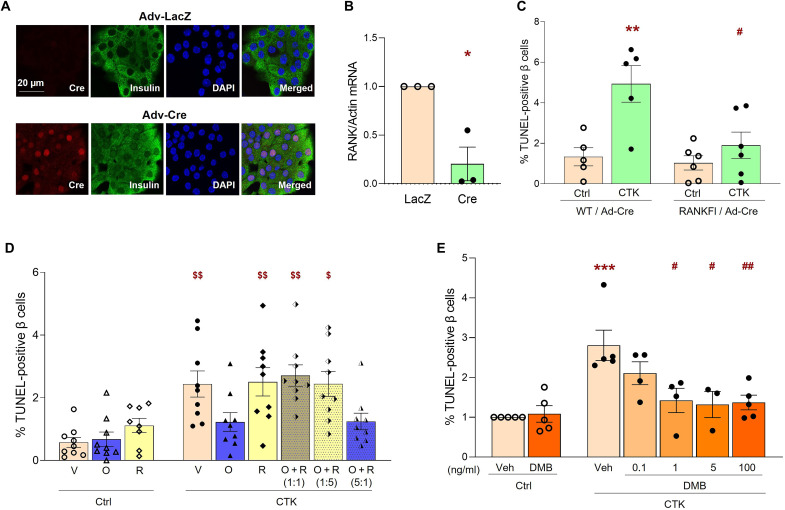
Cytokine-induced rodent and human β cell death require RANKL/RANK. Adv-LacZ– or Adv-Cre–transduced mouse islet cells after 48 hours (**A**) were stained for insulin (green), Cre-recombinase (red), and DAPI (blue), as represented in the individual and merged images (white bar indicates the scale for the images); or (**B**) the ratio of *Rank*/*actin* mRNA was analyzed by real-time qPCR (*n* = 3). *P < 0.05 versus LacZ. (**C**) Percent TUNEL-positive β cells in mouse islet cells from WT or *Rank-floxed* (RANKFl) mice transduced with Adv-Cre for 48 hours and treated without (Ctrl) or with cytokines (CTK) for an additional 24 hours (*n* = 5 to 6). **P < 0.01 versus WT-Ctrl; *^#^P* < 0.05 versus WT-CTK. Percent of TUNEL-positive β cells in human islet cells treated without (Ctrl) or with CTK for 24 hours and (**D**) with Veh (V), 100 ng/ml of hOPG (O), or hRANKL (R) alone, or in combination at a 1:1, 1:5, or 5:1 ratio of OPG/RANKL (O + R), respectively; ^$^*P* < 0.05, ^$$^*P* < 0.01 versus all other groups without symbols, on duplicate samples of *n* = 9; or (**E**) with Veh or DMB (0.1 to 100 ng/ml), with Ctrl-Veh represented as 100% (Ctrl-Veh value 1.54 ± 0.42%; *n* = 4 to 8); ***P < 0.001 versus Ctrl-Veh; *^#^P* < 0.05, *^##^P* < 0.01 versus CTK-Veh. Experiments were done in duplicate with 5 to 10 fields and 1646 ± 124 β cells per sample analyzed for mouse islets (C), and 5 to 10 fields and 1144 ± 167 β cells per sample analyzed for human islets (D and E). Individual symbols in the graphs represent independent experiments on individual mouse or human islet preps, averaging duplicate samples for all experiments (A to E). All data represent means ± SEM. All statistical analysis was by ANOVA with Tukey’s post hoc analysis.

Analysis of the available single-cell RNA sequencing (RNA-seq) datasets of the mouse pancreas (*32*) shows that *Rank* and *Opg* are expressed mainly in β cells with some expression in other cell types (fig. S2, A and B), but *Rankl* is undetectable. Cytokine treatment of INS1 cells (fig. S2C) significantly increased *Rank* expression as early as 8 hours after cytokine treatment measured by quantitative polymerase chain reaction (qPCR). To assess the role of the RANK pathway in proinflammatory cytokine–induced rodent β cell death, the receptor, RANK, was acutely deleted in vitro from mouse islet cells. Islet cells from *Rank-floxed* mice transduced for 48 hours with adenovirus (Adv)–Cre recombinase showed nuclear Cre staining in numerous β cells when costained for Cre and insulin (Fig. 3A), which translated to ~80% reduction of *Rank* mRNA by qPCR compared to Adv-LacZ–transduced cells (Fig. 3B). To assess the effect of *Rank* deletion on cytokine-induced β cell death, Adv-Cre–transduced islet cells from wild-type (WT) and *Rank-floxed* mice were treated with cytokines for an additional 24 hours. Cytokines significantly increased β cell death in WT Adv-Cre–transduced islet cells (4.93 ± 0.9%); however, *Rank-floxed* Adv-Cre–transduced cells were protected (1.9 ± 0.65%) (Fig. 3C). Thus, deletion of *Rank* significantly reduces cytokine-induced, but not basal, β cell death, demonstrating that RANK is essential for cytokine-induced toxicity in rodent β cells.

### RANKL/RANK pathway mediates cytokine-induced human β cell death; OPG and DMB protect against this cytotoxicity

Analysis of single-cell RNA-seq datasets of human pancreata (*33*) shows that *RANK* and *OPG* are expressed in β cells, α cells, ductal and acinar cells, with maximal expression in β cells (fig. S2, D and E). Although *RANKL* is barely detectable in human islets under basal conditions, cytokine treatment for 24 hours significantly increased RANKL secretion from human islets (fig. S2F). We have shown that RANK is required for cytokine-induced rodent β cell death (Fig. 3C) and, therefore, hypothesized that this pathway is important for mediating human β cell death against cytokines. We tested this through competition using varying concentrations of human (h)OPG and hRANKL, alone or in combination, with or without cytokines and assayed for β cell death (Fig. 3D). Basal β cell death (0.6 ± 0.13%) was not significantly altered with the addition of OPG (0.7 ± 0.16%) or RANKL (1.1 ± 0.17%) alone, although there was a tendency toward increased β cell death with RANKL. As expected, cytokine-induced (2.4 ± 0.29%) human β cell death was significantly reduced with 100 ng/ml of OPG (1.2 ± 0.2%) treatment; however, 100 ng/ml of RANKL (2.5 ± 0.33%) had no effect on cytokine-induced human β cell death. When added in combination, RANKL completely obliterated the protective effect of OPG on cytokine-induced β cell death, at a ratio of 1:1 (2.7 ± 0.27%) or 1:5 (2.4 ± 0.28%) of OPG to RANKL, respectively (Fig. 3D). When the ratios were reversed with excess OPG to RANKL (5:1), β cell death was significantly reduced (1.2 ± 0.2%), comparable to levels observed with OPG alone (Fig. 3D). Thus, OPG likely mediates its prosurvival effect on human β cells by inhibiting RANKL/RANK.

On the basis of these findings, we surmised that the osteoporosis drug, DMB, a human RANKL-targeting antibody, should also protect human β cells. The 2.80 ± 0.38-fold increase in cytokine-induced human β cell death was significantly reduced (1.42 ± 0.30 to 1.31 ± 0.33-fold) with DMB (1 to 100 ng/ml) (Fig. 3E), a further validation that the RANKL/RANK pathway is required for cytokine-mediated human β cell death. DMB, like OPG, also reduced cytokine-activated proinflammatory signaling pathways p-NF-κB-Ser536 (Fig. 2, C and D) and p-Stat1-Ser727 (Fig. 2, E and F) in human islets. Collectively, these studies indicate a vital role for RANKL/RANK in cytokine-mediated rodent and human β cell death.

### RANK-TRAF6 interaction is critical for cytokine-induced rodent β cell death and NF-κB activation

**Fig. 4.**
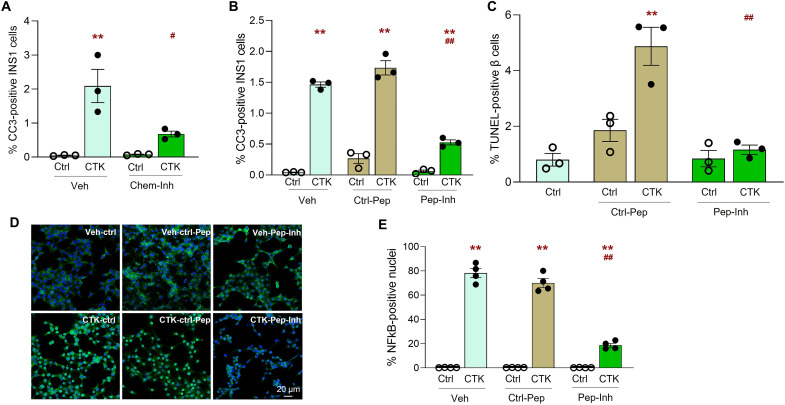
RANK/TRAF6 interaction is required for cytokine-induced β cell death and for NF-κB nuclear translocation. Percent cleaved caspase-3 (CC3)–positive INS1 cells treated without (ctrl) or with CTK for 16 hours and (**A**) with veh or TRAF6 chemical inhibitor (Chem-inh; 5 μM) (*n* = 3) or (**B**) with Veh, control-peptide (ctrl-pep), or TRAF6 peptide inhibitor (Pep-inh; 30 μM) (*n* = 3). (**C**) Percent TUNEL-positive β cells from mouse islet cells treated without (ctrl) or with CTK in the presence of ctrl-pep or Pep-inh for 24 hours (*n* = 3). INS1 cells treated without (ctrl) or with CTK in the presence of Veh, ctrl-pep, or Pep-inh for 30 min (**D**) immunostained for NF-κB (green) and DAPI (blue) and (**E**) quantified for percent NF-κB–positive nuclei (*n* = 4). **P < 0.01 versus ctrl in the same treatment group; ^#^*P* < 0.05, ^##^*P* < 0.01 versus CTK/Veh and CTK/ctrl-pep. Experiments were done in duplicate; with 8 fields and 7678 ± 1208 INS1 cells per sample (A and B), 3 fields and 2898 ± 270 INS1 cells per sample (E), and 5 to 10 fields and 889 ± 96 β cells per sample (C), analyzed. Individual symbols in the graphs represent independent experiments in INS1 cells or individual mouse islet preps, averaging duplicate samples for all experiments (A to E). All data represent means ± SEM. All statistical analysis was by ANOVA with Tukey’s post hoc analysis.

TRAF6 is a key intracellular adaptor that mediates downstream RANKL/RANK signaling (*34*). We tested whether RANK facilitated cytokine-induced β cell death through its interaction with TRAF6. We used two distinct inhibitors of RANK-TRAF6 interaction: (i) the chemical inhibitor compound 6877002, which disrupts TRAF6 interaction with both CD40 and RANK (*35*), and (ii) a TRAF6 peptide inhibitor that binds RANK and thereby specifically inhibits TRAF6 interaction with RANK (*36*). Treatment of INS1 cells with the chemical (Fig. 4A) or peptide (Fig. 4B) TRAF6 inhibitor, but not with vehicle or control peptide, significantly reduced the cytotoxic effects of cytokines on INS1 cells, as assessed by cleaved caspase 3 staining, demonstrating that the interaction of RANK with TRAF6 is required for cytokine-induced β cell death. To determine the relevance of the RANK-TRAF6 interaction in primary mouse β cells, mouse islet cell cultures were treated with either the TRAF6 inhibitor peptide or scrambled control peptide in the absence or presence of cytokines and β cell death assessed by insulin TUNEL costaining. Cytokine-induced β cell death was significantly reduced by the TRAF6 peptide inhibitor but not the control peptide (Fig. 4C), indicating that RANK-TRAF6 interaction is essential for cytokines to be effective in primary β cells. NF-κB activation mediates cytokine-induced β cell death (*37*). Hence, we hypothesized that disrupting RANK-TRAF6 interaction will affect the activation of the NF-κB pathway in β cells. Cytokine treatment of INS1 cells caused nuclear translocation, an indicator of pathway activation, of NF-κB in 78.2 ± 3.83% of the cells (Fig. 4, D and E). Treatment with the TRAF6 peptide inhibitor, but not the control peptide, significantly reduced (18.7 ± 1.63% versus 70.03 ± 3.72%, respectively) nuclear localization of NF-κB (Fig. 4, D and E), demonstrating that RANK-TRAF6 interaction is needed for cytokine-induced NF-κB activation in β cells.

### OPG and DMB reduce production of proinflammatory cytokines in activated human CD4 and CD8 T cells by inhibiting RANKL-induced activation of monocytes

**Fig. 5.**
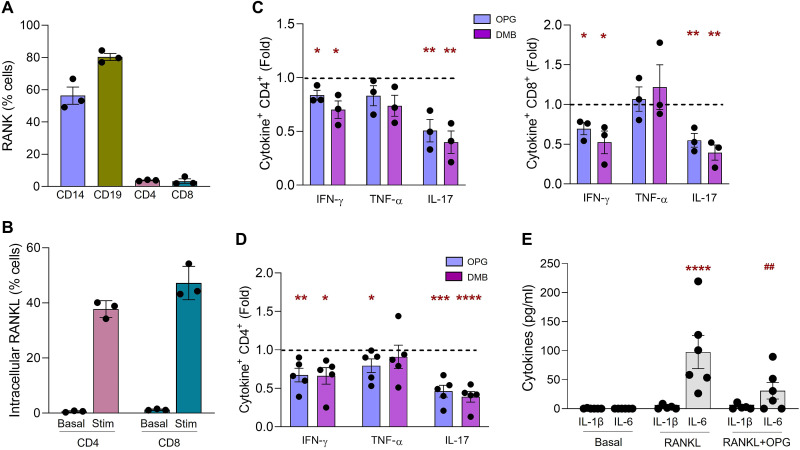
Effects of RANKL, OPG, and DMB on human immune cells. Flow cytometry analysis of (**A**) percent RANK^+^ cells in human PBMCs from healthy blood donors. (**B**) Percent intracellular RANKL-positive CD4 and CD8 T lymphocytes before (Basal) and after (Stim) activation of human PBMCs with plate-bound anti-CD3 (0.5 μg/ml) (*n* = 3). IFN-γ, TNF-α, and IL-17 cytokine production (**C**) in CD4 (left) and CD8 (right) T cells in PBMCs stimulated with plate-bound anti-CD3 (0.5 μg/ml) or (**D**) in CD45RA^+^ naïve CD4 T cells stimulated in the presence of CD14^+^ monocytes (MN) with plate-bound anti-CD3 (0.5 μg/ml) and soluble anti-CD28 (1.0 μg/ml) (*n* = 3 to 6). Cytokines were assessed in the absence (untreated) or presence of 500 ng/ml each of OPG (blue bar) or DMB (pink bar) for 72 hours. Intracellular cytokine production is shown on the *y* axis as the ratio (fold) of % cytokine^+^ cells treated with OPG or DMB versus untreated group. Dotted line (at 1.0) represents no difference in intracellular cytokine production between untreated and treated. *P < 0.05; **P < 0.01; ***P < 0.001; ****P < 0.0001 versus untreated. (**E**) Levels (picograms per milliliter) of IL-1β and IL-6 in supernatants of CD14^+^ MNs incubated with RANKL (100 ng/ml) for 24 hours in the presence or absence of OPG (*n* = 6). ****P < 0.0001 versus basal; ^##^*P* < 0.05 versus RANKL. Individual symbols in the graphs represent independent experiments on different human immune cell donors, averaging triplicate samples for all experiments (A to E). All data represent means ± SEM. Statistical analysis was by *t* test (C and D) and by ANOVA with Tukey’s post hoc analysis (E).

We assessed the levels of RANK and RANKL in the immune cell populations from peripheral blood of healthy blood donors. RANK was detected on the surface of monocytes and B lymphocytes (Fig. 5A), while intracellular RANKL was induced in the CD4 and CD8 T lymphocytes upon activation (Fig. 5B). We evaluated the effect of OPG and DMB on the activation of CD4 and CD8 T lymphocytes in the peripheral blood. Treatment with OPG and DMB significantly reduced production of IFN-γ and IL-17 in CD4 and CD8 T cells from anti-CD3–stimulated PBMCs (Fig. 5C). We then tested the effect of OPG and DMB on purified CD45RA^+^ CD4 T cells, stimulated with anti-CD3/CD28, in the presence or absence of monocytes. The inhibitory effect of OPG and DMB on T cells is mediated by RANK^+^ monocytes since the reduction in proinflammatory cytokine production was seen only when CD4 T cells were cocultured in the presence of monocytes (Fig. 5D), but not in their absence (fig. S3A). The treatment with OPG and DMB does not alter the proliferation of T cells (fig. S3B), only the proinflammatory cytokine profile. Furthermore, treatment of monocytes with RANKL induced IL-6 but not IL-1β production, and the IL-6 stimulation was reduced by OPG treatment (Fig. 5E). Since IL-17 production in T cells is regulated by IL-6 (*38*), our data suggest that the inhibition of IL-17 production from T cells by OPG and DMB is mediated by negative regulation of RANK-RANKL–induced activation of monocytes.

### OPG reduces hyperglycemia and insulitis in NOD/Ltj female mice

**Fig. 6.**
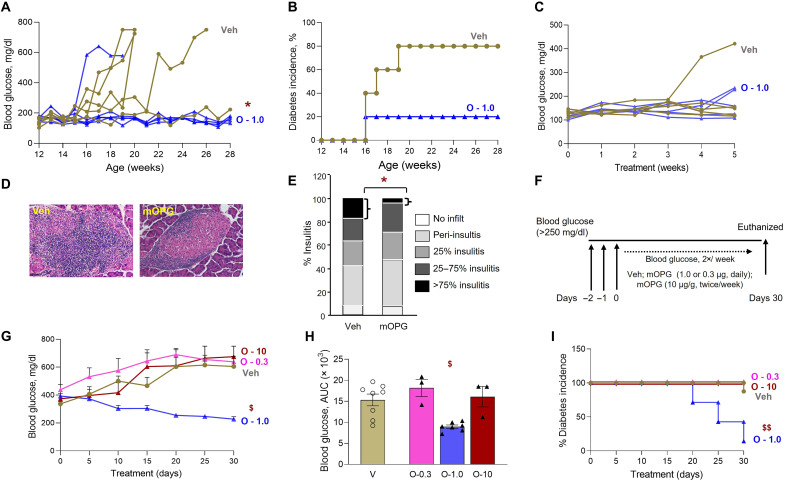
OPG reduces hyperglycemia and insulitis and reverses recent-onset diabetes in NOD/Ltj female mice. Twelve-week-old NOD/Ltj female mice treated every other day with Veh (olive green, circle) or OPG at 1.0 μg/g (blue, triangle) body weight for 16 weeks (*n* = 5 mice per group) were assessed for (**A**) weekly blood glucose; (**B**) percent diabetes incidence (defined as blood glucose >250 mg/dl), no significance between the two groups by log-rank test. Eleven-week-old NOD/Ltj female mice treated daily for 5 weeks with Veh (olive green, circle) or OPG (blue, triangle) at 1.0 μg/g body weight were assessed for (**C**) weekly blood glucose; (**D**) insulitis by H&E staining of pancreatic sections with representative images showing leukocyte infiltration; and (**E**) quantification of percent degree insulitis scored manually on the H&E-stained pancreatic sections using a published formula (*93*), *P < 0.05 for >75% insulitis OPG versus Veh. (**F**) Schematic representation of experimental design for NOD/Ltj female mice with recent-onset diabetes (defined as blood glucose >250 mg/dl for three consecutive days), treated with Veh or OPG at 0.3 μg/g or 1.0 μg/g (daily) or 10.0 μg/g (2×/week), for 30 days, with assessment of body weight and blood glucose every 5 days. NOD/Ltj female mice with recent-onset diabetes treated with Veh (V, olive green, *n* = 8), OPG at 0.3 μg/g (O-0.3, pink, *n* = 3), 1.0 μg/g (O-1.0, blue, *n* = 7), or 10.0 μg/g (O-10, brown, *n* = 3) for 30 days were assessed for (**G**) blood glucose; (**H**) AUC for blood glucose and (**I**) percent diabetes incidence; *P < 0.05 versus Veh; ^$^*P* < 0.05, ^$$^*P* < 0.01 versus all other groups; by mixed model analysis (A and G), by ANOVA with Tukey’s post hoc analysis (H), or by log-rank test (I). Individual symbols in the graphs represent individual mice, and average values represent the average of individual mice in that group. All data represent means ± SEM.

To examine the translational relevance of our in vitro findings, we tested the effect of OPG in NOD/Ltj female mice, a preclinical model of T1D (*26*–*29*). Prediabetic 12-week-old mice were injected every other day with vehicle (control) or OPG at 1.0 μg/g body weight for 16 weeks. This OPG dose was chosen on the basis of its effectiveness in our previous in vivo findings in young, aged, and streptozotocin-treated mice (*12*). The average initial blood glucose (139 ± 12.5 to 158 ± 7.7 mg/dl) was not significantly different between the two groups (Fig. 6A). Four of the five vehicle-treated mice became hyperglycemic (blood glucose >250 mg/dl) between 16 and 20 weeks of age, with blood glucose continuing to rise thereafter. In contrast, only one of the five OPG-treated mice reached hyperglycemia at 16 weeks, with significantly lower average blood glucose compared to vehicle-treated mice (Fig. 6A). Diabetes incidence, defined in these mice as blood glucose ≥250 mg/dl for two consecutive readings (obtained over 10 days), although not significantly different between the two groups due to the small numbers, reached a maximum of 80% by 20 weeks in vehicle-treated mice, whereas it reached a maximum of 20% by 16 weeks in the OPG-treated mice (Fig. 6B). To determine whether continuous OPG treatment was necessary, treatment was stopped after 28 weeks. Diabetes incidence in the OPG-treated group rose to levels similar to vehicle-treated mice, implying that continual OPG treatment was essential.

To examine the effect of OPG on insulitis, the lymphocyte infiltration of islets, prediabetic 11-week-old NOD/Ltj female mice were treated daily for 5 weeks with vehicle or OPG (1.0 μg/g). There were no significant differences in body weight or blood glucose as both groups of mice remained euglycemic, except for one saline-treated mouse that became hyperglycemic by week 4 of treatment (Fig. 6C). Insulitis, assayed by hematoxylin and eosin (H&E) staining (Fig. 6D) and quantified, was similar in most quartiles, except for the most aggressive intra-islet insulitis (>75%), which was significantly reduced in OPG-treated (3.2 ± 2.65) versus control (16.8 ± 3.87) mice (Fig. 6E). To analyze the cellular composition of the infiltrate, we performed three-color immunohistochemical (IHC) staining for specific immune cell markers to identify CD4^+^ and CD8^+^ T cells, regulatory T cells (T_regs_), B cells, and macrophages, as well as insulin to identify β cells in pancreatic sections of the two groups of mice. Except for T_regs_, which were undetectable in both groups, the other immune cell types were detected and seemed similar in vehicle- and OPG-treated mice (fig. S4, A and B).

### OPG reverses recent-onset T1D, improves glucose clearance, and increases plasma insulin in NOD/Ltj female mice

Targets that delay or prevent diabetes in NOD/Ltj mice have not been successful thus far in clinical trials for T1D. On the other hand, targets that reverse T1D in NOD/Ltj mice have shown more clinical promise (*26*–*29*). Therefore, we tested the potential for OPG to reverse T1D in NOD/Ltj mice. NOD/Ltj female mice with spontaneous recent-onset T1D (defined as blood glucose >250 mg/dl for three consecutive days) were treated with vehicle (daily) or different regimens of OPG (0.3 and 1.0 μg/g daily or 10.0 μg/g 2×/week) for 30 days (Fig. 6F). All four groups of mice started out diabetic, with initial average blood glucose ranging from 337 ± 17.4 to 438 ± 37.2 mg/dl (Fig. 6G). Blood glucose rose further in vehicle-treated (605 ± 73.6 mg/dl), low-dose (0.3 μg/g; 638 ± 112 mg/dl), and very high–dose (10.0 μg/g; 675 ± 75 mg/dl) OPG-treated mice by day 30. However, in the 1.0 μg/g OPG–treated mice blood glucose fell from an initial average of 393 ± 12.3 to 247 ± 12.8 mg/dl by day 25 and was maintained below hyperglycemic levels until day 30, with a significant difference from the other three groups when compared by mixed model analysis (Fig. 6G), and when assessed by area under the curve (AUC) (Fig. 6H). Blood glucose of the individual mice in the four groups over 30 days is shown in fig. S4C. All mice in the vehicle, 0.3 μg/g, and 10.0 μg/g OPG–treated groups remained diabetic, with 80 to 100% diabetes incidence throughout the treatment (Fig. 6I). However, there was a significant reduction in diabetes incidence to 20% in the 1.0 μg/g OPG–treated group by day 30 (Fig. 6I).

**Fig. 7.**
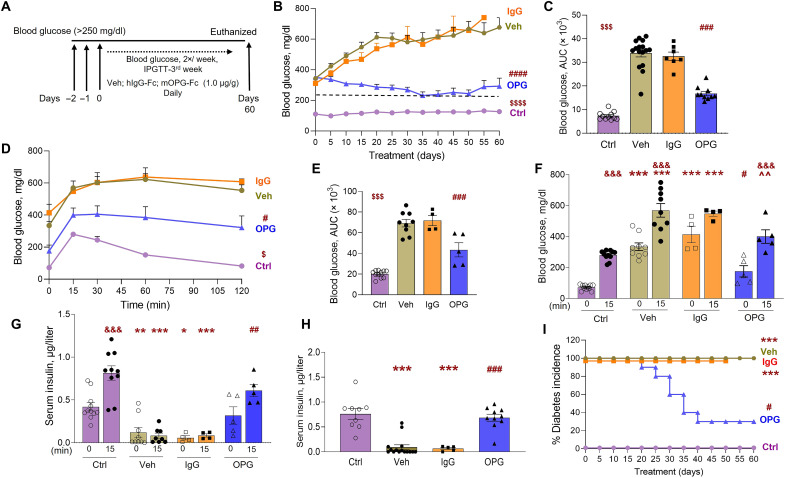
OPG reverses recent-onset T1D, improves glucose tolerance, and increases plasma insulin in NOD/Ltj female mice. (**A**) Schematic representation of experimental design for NOD/Ltj female mice with recent-onset diabetes (defined as blood glucose >250 mg/dl for three consecutive days), treated daily with Veh, IgG (1.0 μg/g), or OPG (1.0 μg/g), for 60 days. Body weight and blood glucose were measured every 5 days, IPGTT with plasma insulin was assessed at week 4, and blood and pancreas were harvested at day 60. NOD/Ltj female control mice that do not develop diabetes (Ctrl, purple, circle, *n* = 11), or mice with recent-onset diabetes treated with Veh (olive green, circle, *n* = 16), IgG (orange, square, *n* = 7), or OPG (blue, triangle, *n* = 10) for 60 days, were assessed for (**B**) blood glucose; (**C**) AUC for blood glucose; (**D**) glucose clearance during an IPGTT (*n* = 4 to 11 per group); (**E**) AUC for IPGTT. ^#^*P* < 0.05, ^###^*P* < 0.001, ^####^*P* < 0.0001 versus Veh and IgG; ^$^*P* < 0.05, ^$$$^*P* < 0.001, ^$$$$^*P* < 0.0001 versus all other groups; by mixed model analysis (B and D) or by ANOVA with Tukey’s post hoc analysis (C and E); (**F**) blood glucose and (**G**) plasma insulin at 0- and 15-min time points during the IPGTT; *P < 0.05, **P < 0.01, ***P < 0.001 versus Ctrl at the same time point; ^#^*P* < 0.05, ^##^*P* < 0.01 versus Veh and IgG at the same time point; ^^*P* < 0.01 versus Veh at the same time point; and ^&&&^*P* < 0.001 versus 0 min of the same treatment; (**H**) plasma insulin at day 60; (**I**) percent diabetes incidence; ***P < 0.001 versus Ctrl; ^#^*P* < 0.05, ^###^*P* < 0.001 versus Veh and IgG; by ANOVA with Tukey’s post hoc analysis (F, G, and H) or by log-rank test (I). Individual symbols in the graphs represent individual mice, and average values represent the average of individual mice in that group. All data represent means ± SEM.

As reversal of diabetes in the 1.0 μg/g OPG–treated group occurred toward the end of the treatment (Fig. 6I), a longer 60-day study was performed. The study was conducted at two different institutions (Mount Sinai, NY and City of Hope, CA) to negate any effects of animal housing on the NOD/Ltj mouse phenotype. In the second cohort done at City of Hope, we included hIgG-Fc as an additional control to account for any effects of the Fc fragment in the recombinant OPG-Fc protein. Spontaneous recent-onset T1D NOD/Ltj female mice were treated daily with vehicle, 1.0 μg/g of OPG, or IgG and subjected to a glucose challenge at days 26 to 28 of treatment (Fig. 7A). Blood glucose in vehicle- and IgG-treated mice rose from 343.94 ± 10.61 and 310.14 ± 16.17 mg/dl at day 0 to 676.14 ± 63.83 and 644 mg/dl at day 60, respectively. In contrast, in OPG-treated mice, blood glucose fell from 349.7 ± 12.9 mg/dl at day 0 to 292.4 ± 52.71 mg/dl at day 60 (Fig. 7B). Blood glucose of the individual mice in the four groups over 60 days is shown in fig. S4D. Blood glucose in the OPG-treated mice was significantly reduced compared to vehicle- and IgG-treated mice but remained higher than in the control ND group, as assessed by mixed model analysis (Fig. 7B), and as corroborated by AUC (Fig. 7C). To further assess glucose homeostasis in these mice, the animals were challenged with an intraperitoneal glucose tolerance test (IPGTT) at days 26 to 28. Glucose clearance in the OPG-treated mice significantly improved compared to the vehicle- and IgG-treated mice but was not completely normalized to levels observed in the control ND mice (Fig. 7D; individual mouse glucose values in fig. S4E), as also indicated by the AUC (Fig. 7E). Fasting plasma glucose at time 0 of the IPGTT was significantly reduced in the OPG-treated (176 ± 36.7 mg/dl) versus vehicle-treated (334.7 ± 24.9 mg/dl) and IgG-treated (414.25 ± 53.55 mg/dl) mice, and was not significantly different, although higher, than the control ND mice (72.27 ± 5.26 mg/dl) (Fig. 7F). The increase in blood glucose 15 min after glucose injection was significant in the control ND, vehicle-, and OPG-treated mice, relative to their fasting blood glucose levels, but was not significantly increased in the IgG-treated mice. The 15-min blood glucose in the OPG-treated mice (400.6 ± 43.6 mg/dl) was significantly reduced compared to the vehicle-treated mice (569.44 ± 43.95 mg/dl) but was not significantly different from IgG-treated (548.5 ± 16.63 mg/dl) and control ND (280.27 ± 9.53 mg/dl) mice (Fig. 7F). Serum insulin measured at the 0- and 15-min time points of the IPGTT showed a significant decrease in fasting and 15-min insulin in the vehicle- and IgG-treated mice, but not the OPG-treated mice, compared to control ND mice (Fig. 7G). The 15-min insulin in the OPG-treated mice (0.61 ± 0.07 ng/ml) was significantly higher than vehicle-treated (0.07 ± 0.03 ng/ml) and IgG-treated (0.085 ± 0.019 ng/ml) mice (Fig. 7G). Similarly, the serum insulin measured at the end of the 60-day treatment showed a significant decrease in the vehicle-treated (0.093 ± 0.048 ng/ml) and IgG-treated (0.066 ± 0.021 ng/ml) groups compared to control (0.762 ± 0.107 ng/ml) and OPG-treated (0.686 ± 0.07 ng/ml) mice (Fig. 7H). Together, the data indicate that OPG treatment increases and maintains normal serum insulin levels in the mice, thus improving hyperglycemia. Diabetes incidence, which was 100% at day 0 for the three groups, remained 100% in vehicle- and IgG-treated mice but dropped to 30% by day 40 in OPG-treated mice and was significantly reduced compared to vehicle- and IgG-treated groups (Fig. 7I) but was not significant compared to the control ND group. Table S1 summarizes the experimental treatments and outcomes for the glucose homeostasis analysis in Figs. 6 and 7.

### OPG treatment significantly increases β cell mass and proliferation in recent-onset T1D mice while maintaining normal β cell survival

**Fig. 8.**
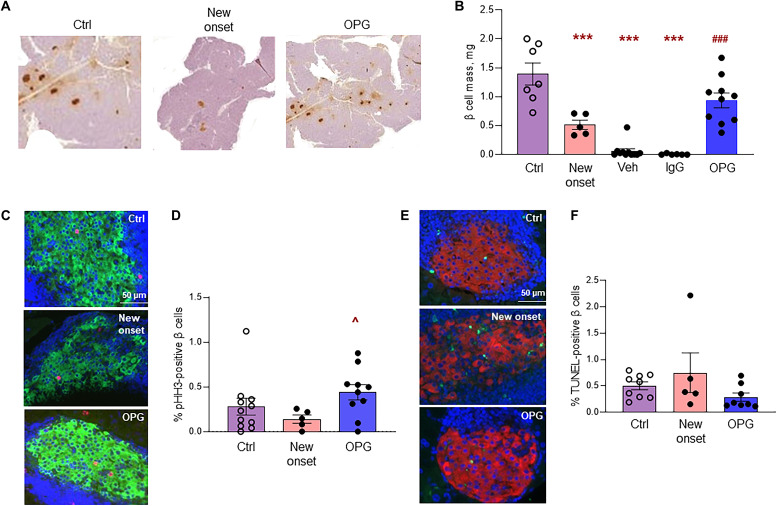
OPG treatment significantly increases β cell mass and proliferation in recent-onset T1D mice while maintaining normal β cell survival. Pancreata from NOD/Ltj female mice with recent-onset diabetes defined as blood glucose >250 mg/dl for three consecutive days were harvested immediately (new onset, *n* = 5) or after 60 days of daily treatment with vehicle (Veh, *n* = 10), 1.0 μg/g IgG (*n* = 6), or 1.0 μg/g OPG (*n* = 10), or from control ND NOD/Ltj female mice of similar age (Ctrl, *n* = 7), and (**A**) stained for insulin as shown in the representative images; (**B**) assessed for β cell mass, ***P < 0.001 versus Ctrl; ^###^*P* < 0.001 versus Veh and IgG, by ANOVA with Tukey’s post hoc analysis; (**C**) stained for insulin (green), pHH3 (red), and DAPI (blue); (**D**) quantified for percent pHH3-positive β cells (*n* = 5 to 11), ^*P* < 0.05 versus new onset by ANOVA with Tukey’s post hoc analysis; (**E**) stained for insulin (red), TUNEL (green), and DAPI (blue); and (**F**) quantified for percent TUNEL-positive β cells (*n* = 5 to 9). The bar indicates the magnification scale for the images. Individual symbols in the graphs represent individual mice. All data represent means ± SEM.

At the end of the treatment, pancreata were harvested from the mice, and β cell mass was quantified on insulin-stained pancreatic sections (Fig. 8A). An additional control group, new-onset diabetic mice was added, in which pancreata were harvested immediately after three consecutive days of hyperglycemia (blood glucose >250 mg/dl) to assess β cell mass before initiation of the treatment. There was a significant ~3-fold decrease in β cell mass in the new-onset diabetic mice (0.518 ± 0.081 mg) compared to the control ND mice (1.394 ± 0.19 mg) (Fig. 8B). After 60 days of treatment with vehicle or IgG, β cell mass was reduced further to almost undetectable levels, 0.062 ± 0.046 and 0.007 ± 0.005 mg, respectively (Fig. 8B). However, 60 days of OPG treatment significantly increased β cell mass (0.939 ± 0.128 mg) compared to the vehicle- and IgG-treated groups and, notably, normalized β cell mass to levels close to control ND (1.394 ± 0.19 mg) mice (Fig. 8B). Percentage of proliferating β cells, assessed by pHH3-insulin costaining (Fig. 8C), was ~2-fold lower in new-onset diabetic (0.141 ± 0.046%) versus control (0.279 ± 0.094%) mice and was significantly higher by ~3-fold in OPG-treated (0.440 ± 0.086%) versus new-onset diabetic mice (Fig. 8D). β cell death, assessed by TUNEL-insulin costaining (Fig. 8E), although not significantly different between the three groups [control (0.502 ± 0.07%), new-onset diabetic (0.75 ± 0.374%), and OPG-treated (0.283 ± 0.076%)], tended to be lowest in the OPG-treated mice (Fig. 8F). As most of the mice in the vehicle- and IgG-treated groups had lost their β cells (Fig. 8B), β cell proliferation and death could not be assessed in these groups.

### Serum from patients with T1D induces human β cell death and dysfunction, but not α cell death; OPG and DMB protect against this cytotoxicity and improve function

**Fig. 9.**
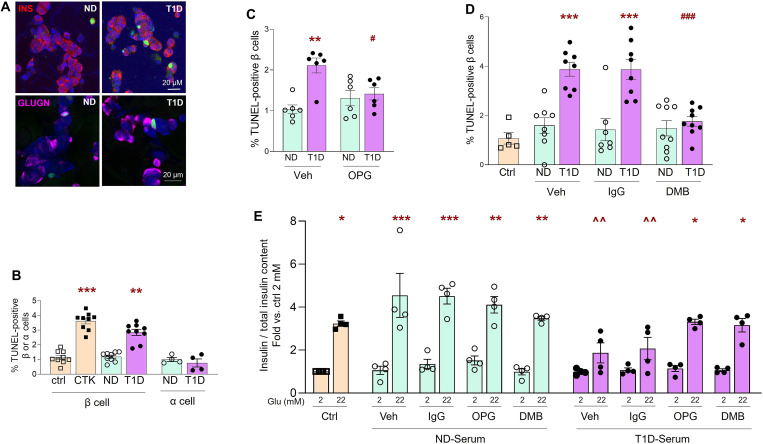
Serum from patients with T1D induces β cell cytotoxicity and dysfunction in human islets, but not α cell cytotoxicity; OPG and DMB protect against this cytotoxicity and improve function. Human islet cells (*n* = 7 preps) cultured for 24 hours in islet media (ctrl), treated with cytokines (CTK), or in media in which the FCS was substituted with serum (10% v/v) from T1D or control ND donors (*n* = 9 each), were (**A**) stained for TUNEL (green), DAPI (blue), and insulin (red) (top) or glucagon (far red) (bottom), as shown in the representative confocal images (bar indicates the scale for the images); and (**B**) quantified for percent TUNEL-positive β cells (*n* = 9) or α cells (*n* = 4). Percent TUNEL-positive β cells in human islet cells (*n* = 3 to 5 preps) cultured for 24 hours in ctrl media or in medium containing serum from either T1D or ND (*n* = 6 to 9 each) subjects and treated with (**C**) either Veh or OPG (100 ng/ml) or (**D**) either Veh, IgG (100 ng/ml), or DMB (100 ng/ml); **P < 0.01, ***P < 0.001 versus Ctrl or ND of the same treatment group; ^#^*P* < 0.05, ^###^*P* < 0.001 versus Veh-T1D and IgG-T1D. (**E**) Secreted insulin (nanograms per milliliter)/total insulin content from human islets (*n* = 2 preps) at 2.2 and 22.2 mM glucose for 45 min, after culturing for 24 hours in regular media (ctrl) or in media containing ND or T1D serum (*n* = 4 each) in the presence of Veh, 100 ng/ml of IgG, OPG, or DMB, represented as fold over ctrl at 2.2 mM (insulin secretion/total insulin content at 2.2 mM in ctrl islets is 0.096 ± 0.03 ng/liter); *P < 0.05, **P < 0.01, ***P < 0.001 versus 2.2 mM of the same treatment group; ^^*P* < 0.01 versus 22.2 mM of Veh and IgG of ND serum. Five to ten fields and 1093 ± 199 β cells per sample in duplicate (A to D) were analyzed. Individual symbols in the graphs represent individual human serum samples, averaging duplicate samples for all experiments (A to E). All data represent means ± SEM. All statistical analysis was by ANOVA with Tukey’s post hoc analysis.

Prior literature shows that serum from T1D subjects induces DNA fragmentation in whole rodent islets or rodent β cell lines (*39*, *40*). To establish an in vitro model of β cell death that has relevance to disease pathology, we assessed whether human T1D serum is cytotoxic to human β cells. Dispersed human islet cells were analyzed for β cell death by insulin-TUNEL costaining (Fig. 9A), after 24 hours of culture in control media containing 10% FCS, or in media in which the FCS was substituted with 10% human serum, either from consented adult T1D subjects with disease diagnosis duration of 1 to 5 years or from ND control subjects best-matched for sex, age, and ethnicity (table S2). Percent human β cell death was comparable when cultured in medium containing FCS (1.14 ± 0.15%) or control ND human serum (1.19 ± 0.11%) (Fig. 9B). In contrast, human β cell death was significantly increased in medium containing T1D serum (2.84 ± 0.21%), similar to that observed with proinflammatory cytokines (3.61 ± 0.20%) used as a positive control for β cell death (Fig. 9B). In T1D, the pancreatic β cell but not the α cell is susceptible to cell death (*41*–*43*). To determine the validity of the T1D serum–induced cytotoxicity to human disease, we tested whether the T1D serum was cytotoxic to α cells. The same human islet cell cultures were simultaneously assayed for α cell death by glucagon and TUNEL costaining (Fig. 9A). Unlike what we observed in β cells, there was no difference in α cell death when cultured with T1D (0.76 ± 0.27%) or ND (0.99 ± 0.13%) human sera (Fig. 9B). These findings suggest that T1D serum–induced human β cell death is a relevant in vitro model for human disease.

To evaluate the effect of OPG and DMB in this model, we first examined the levels of RANKL and OPG in the serum from T1D and ND subjects using an enzyme-linked immunosorbent assay (ELISA). We did not observe any significant difference in the levels of these proteins in the sera from the two groups (fig. S5A). We tested whether T1D or ND serum could affect the expression of *RANKL*, *RANK*, and *OPG* by qPCR in human islets. There was no significant difference in expression of the three genes in human islets treated with either T1D or ND serum (fig. S5B). To determine whether OPG and DMB could protect human β cells against T1D serum–induced cytotoxicity, dispersed human islet cells cultured in medium containing FCS, human T1D, or ND serum were treated with either vehicle or IgG as controls, and OPG (Fig. 9C) or DMB (Fig. 9D), and assayed for β cell death after 24 hours. Elevated human β cell death observed in medium containing T1D serum (2.1 ± 0.18%) relative to ND serum (1.0 ± 0.11%) was significantly reduced with OPG treatment (1.4 ± 0.15%); however, OPG had no effect on basal β cell death in ND serum (1.3 ± 0.19%)–containing medium (Fig. 9C). Similarly, DMB (1.76 ± 0.19%) significantly reduced T1D serum–induced β cell death relative to vehicle (3.88 ± 0.28%) or IgG-treated (3.87 ± 0.40%) cells (Fig. 9D). As in the case of OPG, DMB had no effect on basal cell death observed with ND serum.

β cell dysfunction is another early hallmark of T1D (*3*, *44*). We examined the effect of T1D and ND serum on human β cell function by GSIS. T1D serum, but not ND serum, caused a significant reduction in GSIS (Fig. 9E). Simultaneous treatment with either vehicle, IgG, OPG, or DMB showed that OPG and DMB treatments did not affect the normal basal β cell function observed with ND serum (Fig. 9E). However, the impaired β cell function observed with T1D serum treatment of human islets was significantly improved in the presence of OPG and DMB, but not with vehicle or IgG treatment (Fig. 9E). Total insulin content and insulin stimulation index for these conditions did not change as shown in fig. S5 (C and D). Thus, OPG and DMB protect human β cells and improve their function against the toxicity induced by serum from patients with T1D.

## DISCUSSION

This study identifies RANKL/RANK as a critical mediator of cytokine-induced cell death in rodent and human β cells and reveals the importance of RANK-TRAF6 interaction in cytokine-mediated cytotoxicity. Protection of primary β cells by OPG and DMB against cell death and dysfunction, induced by cytokines and serum from patients with T1D, underscores the clinical relevance of this pathway in T1D. Induction of inflammatory cytokines by RANKL in monocytes and reduction by OPG and DMB in activated T cells from human PBMCs imply the involvement of RANKL/RANK in the immune response. The reversal of recent-onset T1D in female NOD/Ltj mice with OPG treatment suggests that this pathway may have therapeutic potential for T1D.

OPG inhibits cytokine-induced cell death and modulates the p38 pathway in rat β cells (*11*, *22*). Our study unequivocally demonstrates the prosurvival role of OPG and DMB in primary mouse and human β cells in a T1D milieu, which includes proinflammatory cytokines and T1D patient serum. OPG is a decoy receptor that can bind two ligands, RANKL and TNF-related apoptosis-inducing ligand (TRAIL), thereby inhibiting the interaction of these ligands with their respective membrane-bound receptors, RANK and death receptor (*6*–*9*). We focused on the RANKL/RANK pathway in the prosurvival effect of OPG in β cells against cytokines for several reasons. (i) RANKL/RANK can induce apoptosis in various cell types, including monocytes, osteoclast precursors, and cancer cells (*17*–*21*), implying a similar proapoptotic role in β cells. (ii) There is an interdependence of the TNF-α and RANKL signaling pathways; each can induce the expression of the other in monocytes and osteoclasts, there are epitopes that are common to both ligands, and there are common downstream molecules that interact with both pathways (*45*–*49*). (iii) Of the two OPG ligands, TRAIL is typically associated with inducing cell death. However, TRAIL causes death mainly in tumor cells and not in healthy cells (*50*). (iv) RANKL is more sensitive to inhibition by OPG than TRAIL as a 1:1 ratio of OPG to RANKL is sufficient to block activity of RANKL, whereas a 10:1 ratio of OPG to TRAIL is required to block TRAIL activity (*51*). Using three independent approaches, genetic deletion of RANK, competition of OPG and RANKL, and inhibition of RANKL with DMB, we show that RANKL/RANK is essential for cytokine-induced rodent and human β cell death. Addition of RANKL to the cytokine mix did not further increase human β cell death (Fig. 3D), suggesting that the cell death response with cytokines is maxed out. The RANKL/RANK pathway is also important in T1D serum–induced human β cell death, as the RANKL antibody DMB can protect against this cytotoxicity. Using a specific inhibitor to disrupt RANK-TRAF6 binding, we showed that interaction of RANK with TRAF6 was critical for cytokine-mediated β cell death and for the activation of the NF-κB pathway. OPG and DMB down-regulated two key proinflammatory signaling pathways activated by cytokines, NF-κB and STAT1, in mouse islets and primary human β cells. Mechanistically, we have shown that RANK, through its interaction with the adaptor molecule TRAF6, plays a vital role in cytokine-mediated β cell death.

Knowing that OPG and DMB can induce proliferation (*12*) and protect β cells (Figs. 1 to 3), we wanted to examine their effect on β cell function. Our in vitro GSIS studies in mouse islets show that OPG does not affect β cell function under basal conditions. However, OPG significantly improved and normalized the impaired GSIS observed under cytokine stress in mouse islets. Similarly, human islets treated with T1D serum, but not control ND serum, showed reduced GSIS; both OPG and DMB, but not IgG, improved human β cell function. Thus, OPG and DMB can improve rodent and human β cell function under stress, as shown previously with OPG in rodent islets (*22*).

To control for the effects of the Fc fragment of the recombinant OPG-Fc or DMB antibody used in our studies, we used IgG as an additional control in every category of experiments in vitro, including cell death assay, Western blot analysis, and GSIS assays, as well as for the in vivo diabetes reversal studies. Consistently, IgG effects phenocopied that of the vehicle control, indicating that the beneficial effects observed in the studies are mediated by OPG or DMB, and not the Fc fragment.

RANKL/RANK/OPG are dysregulated not only in T2D (*52*, *53*) and gestational diabetes (*54*, *55*) but also in T1D. Polymorphism in the OPG gene may be linked to T1D (*56*). Plasma OPG and total (soluble and free) RANKL change in patients with T1D compared to controls, implying a dysregulation of this pathway in T1D (*57*–*62*). Although the levels of these proteins change in the serum of patients with T1D, there is little consensus of whether they increase or decrease. It seems to depend on the stage of the disease, with wide variation between studies. Serum RANKL levels were found to remain unchanged (*57*), decrease (*58*), or increase (*59*–*60*) in T1D versus control subjects. Most studies show an increase in OPG in T1D subjects (*57*, *58*, *60*–*62*); however, one study showed a decrease in OPG with recent-onset diabetes (*59*). A recent study showed increased levels of circulating OPG in newly diagnosed T1D subjects, which decreased with disease progression (*58*). In the small cohort that we examined, the levels of RANKL and OPG were not significantly different between ND and T1D subjects. Circulating OPG levels in T1D subjects, although changing, are much lower than the concentration we have used to treat T1D, or what has been used for treating bone resorption (*63*), in mice. Besides changes in RANKL/OPG levels, several cytokines (both pro- and anti-inflammatory) and chemokines are also increased in the blood from T1D subjects compared to controls, as shown by several serum proteomic studies (*64*–*67*). It is possible that the increased proinflammatory cytokines/chemokines in T1D serum could partially mediate its cytotoxic effects on β cell health. What specific components in T1D serum induce β cell cytotoxicity and dysfunction is of great interest and is being pursued by many in the field including us.

Our in vitro studies on human PBMCs show that (i) RANKL is almost undetectable basally but gets highly induced in activated T cells; (ii) RANKL stimulates the proinflammatory cytokine IL-6, but not IL-1β, in monocytes. IL-6 is a key upstream regulator for the differentiation of T helper 17 (T_H_17) cells (*68*), inducing inflammation and β cell cytotoxicity (*38*, *69*); (iii) OPG and DMB significantly reduce inflammatory cytokines IL-17 and IFN-γ, both known to stimulate the autoimmune response in T1D (*69*), in activated CD4^+^ and CD8*^+^* T cells, but only in the presence of monocytes. It is likely that activated T cells, through an increase in RANKL, could stimulate IL-6 production in monocytes, which would direct T cells toward the T_H_17 phenotype. The relevance of this in the context of T1D will be examined in future studies.

Our in vivo findings show that OPG significantly reduces hyperglycemia and insulitis in prediabetic NOD/Ltj female mice, a preclinical model of T1D. Although OPG treatment reduced diabetes incidence, the reduction was not significant, likely due to the small cohort size. Relevant to disease treatment, OPG could reverse recent-onset T1D in NOD/Ltj female mice significantly by 70%. NOD mice are extremely sensitive to their environment and treatment in terms of disease incidence (*26*–*29*). Our reversal studies performed in two different institutions (Mount Sinai, NY and City of Hope, CA) gave very similar results, further strengthening the conclusions of this study. The inclusion of an additional IgG-Fc treatment control, to offset any effects from the Fc fragment of recombinant OPG, showed similar outcomes to the saline control. We found significant improvement in blood glucose, plasma insulin, and glucose tolerance, resulting in a reversal of diabetes in OPG-treated versus saline- or IgG-treated diabetic NOD mice. This was reflected in their β cell mass at the end of the 60-day treatment. The OPG-treated mice had significantly increased β cell mass compared to both saline and IgG controls, which had almost no remnant β cells. The β cell mass in the new-onset diabetic mice was significantly reduced compared to ND control mice, but the OPG-treated mice seemed to have recovered their β cell mass as it was not significantly different from ND control mice. β cell proliferation showed a similar pattern, where OPG-treated mice showed a significant increase compared to new-onset diabetic mice. There were no significant differences in β cell death, although there was a trend toward a decrease in OPG-treated mice. We observed increased serum insulin in the OPG-treated mice during the IPGTT and at the end of the treatment compared to saline- and IgG-treated mice. This likely reflects increased β cell mass, improved β cell function, or both in the OPG-treated mice. Our in vitro GSIS studies show that both OPG and DMB enhanced GSIS in rodent and human islets under stress, suggesting that OPG treatment in diabetic NOD mice could likely improve β cell function.

Reversal of recent-onset T1D was dependent on the regimen of OPG treatment. Daily administration of 1.0 μg/g, but not 0.3 μg/g, OPG reverted T1D. Unexpectedly, the higher 10.0 μg/g dose administered 2×/week did not reverse T1D. One plausible interpretation is that daily treatment is required for reversing T1D. However, this seems unlikely considering recombinant OPG has far greater stability than native OPG with a half-life in days versus minutes, respectively (*70*). An alternate explanation is that at the higher, 10.0 μg/g, dose, OPG-Fc also inhibits TRAIL [as 10× more OPG is required to inhibit TRAIL versus RANKL (*51*)], and TRAIL has beneficial effects in the context of T1D (*71*). A regimen of 5 to 10 μg/g OPG in rodents caused increased bone mass within 2 to 4 weeks (*63*), implying a different dose/duration requirement of OPG for improving bone versus β cell health.

An important and justified question raised in the quest for T1D therapies is the relevance of NOD/Ltj mouse studies and their translation to successful interventions in T1D clinical trials. Historically, there have been many failures in this bench-to-bedside translation (*26*–*29*). Most treatments that were successful in NOD/Ltj mice for either early prevention (treatment initiated at 3 to 4 weeks of age) and/or late prevention (treatment initiated at 10 to 12 weeks of age) failed to show efficacy in clinical trials (*26*–*29*). Few therapies have been tested for their ability to reverse T1D in NOD/Ltj mice with new-onset diabetes, and fewer still with established disease. Most of these have been immunotherapies, some of which have shown efficacy in clinical trials, implying that properly controlled reversal studies in NOD/Ltj mice can be a good preclinical model for T1D therapeutics. Our current study meets all the major criteria recommended for the NOD/Ltj mouse to be a good preclinical model (*26*–*29*), including testing the reagent on sufficient numbers of mice at two different locations, using placebo (IgG) controls other than saline treatment, initiating treatment after three consecutive days of blood glucose >250 mg/dl, reversal of diabetes defined as blood glucose <250 mg/dl for two consecutive weeks, and age of spontaneous diabetes falling between 17 and 20 weeks of age on average. Our study is one of a handful to test a therapeutic that targets the β cell rather than only the immune system for its ability to reverse T1D in NOD/Ltj mice. Approaches to alter the pathologic prone β cell microenvironment are especially relevant in our current thinking that β cells play a central role in eliciting proinflammatory signaling and T1D development (*3*, *72*).

Our finding that OPG reverses early-onset T1D, and reduces severe insulitis, suggests that OPG regulates not only β cells but also immune cells in T1D. This is highly probable considering that the RANKL/RANK pathway is active in innate and adaptive immune cells, and RANKL and TRAIL, targets of OPG, have multiple effects on the immune system (*17*–*21*). Our findings in human PBMCs suggest that the RANK pathway stimulates the T_H_17 phenotype, which could affect the autoimmune response in T1D. In our in vivo studies, although we found a significant decrease in severe insulitis with OPG treatment, we did not observe any obvious differences in the immune cell composition of islet infiltrates in vehicle- versus OPG-treated NOD mice. One explanation could be the late initiation of OPG treatment, at 11 weeks of age, when insulitis is already well established in NOD mice. Another possibility is that OPG treatment could likely affect the function and phenotype rather than the composition of immune cells, directing them toward a less inflammatory phenotype, as suggested by our in vitro human immune cell studies. There is increasing evidence in other autoimmune diseases (multiple sclerosis, rheumatoid arthritis, and inflammatory bowel disease) that the RANKL/RANK pathway is involved in the induction of a proinflammatory phenotype of immune cells and exacerbation of the disease (*73*–*78*). Inhibition of the RANKL/RANK pathway ameliorates inflammation through multiple mechanisms, including affecting innate immune cells, natural killer cells, B cells, and T cells. Currently, DMB is in phase 2 clinical trials for the treatment of the autoimmune disease rheumatoid arthritis (*76*), not only due to its effect on bone but also because it directly modulates the inflammatory response (*77*).

A valid concern with the systemic use of OPG or DMB is their potential effects on multiple target tissues, such as the β cell, bone, immune cells, hepatocytes, muscle, fat, mammary gland, and brain (*6*–*9*). There is evidence that the quality of bone deteriorates with higher fracture risk in patients with T1D (*58*, *60*, *61*, *78*). It is reassuring that the effects of OPG and DMB on the β cell, hepatocytes, muscle, fat, and bone are beneficial in the context of diabetes, as they enhance β cell growth (*12*, *13*), function (*22*, *54*), and survival (*11*, *22*); improve hepatocyte, muscle, and adipose insulin sensitivity (*52*, *79*–*81*); and augment bone formation (*6*–*9*, *14*–*16*). Increased systemic levels of OPG in transgenic rats and mice do not have obvious deleterious effects on their immune or mammary gland function (*82*–*84*). DMB, an FDA-approved drug, has been used to treat osteoporosis for more than a decade, is approved for the treatment of skeletal adverse effects in patients with cancer, and is currently in clinical trials for rheumatoid arthritis and in children with cancer despite its multi-organ actions, testament to its relative safety (*14*–*16*, *76*, *85*, *86*).

Our current and previous (*12*) studies demonstrate that OPG and the anti-osteoporotic drug DMB through inhibition of a common pathway, RANKL/RANK, can enhance the three essential cellular processes, proliferation, function, and survival, in rodent and human β cells and reduce proinflammatory function of human immune cells. The clinical and therapeutic potential of targeting the RANKL/RANK pathway in T1D is underscored by our observation that OPG and DMB protect and enhance the function of human β cells against human T1D serum–induced cytotoxicity and that OPG can reverse T1D in NOD mice. Future studies will assess the potential of OPG or the repurposing of DMB for the treatment of T1D, either alone or in combination with other therapies that target autoimmunity.

## MATERIALS AND METHODS

### Animal studies

For in vitro studies, islets were isolated from the following strains, age, and sex of mice. For Western blot analysis, 4- to 6-month-old male and female C57BL/6 mice (the Jackson Laboratory, Bar Harbor, ME); for cell death assays, 2- to 6-month-old male C57BL/6 mice; for Adv-LacZ and Adv-Cre transductions, 4- to 6-month-old male and female *Rank-floxed* and WT littermate mice on a C57BL/6 background; and for GSIS, 4- to 6-month-old male and female C57BL/6 mice were used. For in vivo studies, female NOD/ShiLtJ mice (the Jackson Laboratory) ranging from 11 to 30 weeks of age were used. Prediabetic NOD/Ltj mice at 11 to 12 weeks of age were injected subcutaneously with vehicle [saline or phosphate-buffered saline (PBS)] or 1.0 μg/g body weight of OPG, either every other day for 16 weeks or daily for 5 weeks. Body weight and blood glucose were measured twice per week. To assess whether OPG can reverse T1D, female NOD/Ltj mice with spontaneous recent-onset diabetes, defined as blood glucose >250 mg/dl for three consecutive days, were injected subcutaneously with either saline or OPG at 0.3 or 1.0 μg/g body weight, daily, or OPG at 10.0 μg/g body weight, twice a week, for 30 days. A similar separate cohort of recent-onset diabetic mice was injected daily with either saline, IgG (human IgG Fc fragment) as control, or OPG at 1.0 μg/g body weight for 60 days. Some of the vehicle- and IgG-treated mice were euthanized before the 60-day time point based on the body score guidelines and animal welfare policy. However, all the OPG-treated mice completed the 60-day study. Two separate cohorts of mice for vehicle and OPG treatment were tested at two different animal facilities (Icahn School of Medicine at Mount Sinai, New York, NY and City of Hope, Duarte, CA). A cohort of mice for each treatment was subjected to IPGTT at 26 to 28 days of treatment. Blood samples were collected through facial vein before and after 15 min of glucose injection to measure serum insulin. Pancreata and serum were harvested at the end of the study. Animal studies were performed with the approval of, and in accordance with, guidelines established by both Icahn School of Medicine at Mount Sinai, NY and Beckman Research Institute, City of Hope, CA, and principles of laboratory animal care were followed.

### Peptides

Recombinant OPG-Fc (referred to as OPG) protein was used in a species-specific manner for these studies. Human (h)OPG-Fc (805-OS) and mouse (m)OPG-Fc proteins (459-MO) were procured from R&D Systems (Minneapolis, MN) for the in vitro and in vivo experiments. The Fc portion of human and mouse OPG-Fc is derived from human IgG-Fc. hRANKL peptide (ALX-522-012) was obtained from Enzo Life Sciences (Farmingdale, NY). DMB (Prolia) was purchased from Amgen (Thousand Oaks, CA). Human IgG-Fc (referred to as IgG) fragment was obtained from Sigma-Aldrich (St. Louis, MO). The vehicle to reconstitute these peptides was PBS with 0.1% bovine serum albumin (PBSA), saline, or DMB (Prolia) vehicle.

### Mouse and human islet cell culture; Adv-transduction

Mouse islets were isolated by collagenase digestion and histopaque gradient separation and cultured in complete medium (RPMI 1640 with 5.5 mM glucose, 10% FCS, and 1% penicillin-streptomycin). Human islets (table S3) were obtained from the Integrated Islet Distribution Program, Prodo Laboratories, and Southern California Islet Resource Center. Rodent and human islets were cultured in complete media for at least 24 hours before they were handpicked under the microscope for treatment and analysis. Islet cell cultures were prepared by trypsinization for 10 min with intermittent pipetting. Cells from 50 to 70 islet equivalents (IEQs; 1 IEQ = 125 μm diameter) were plated on coverslips in a 24-well plate initially in a small volume (50 μl) of media for 2 hours to allow attachment. Trypsinized *Rank-floxed* or WT islet cell cultures were transduced with recombinant Adv-LacZ or Adv-Cre recombinase (Gene Transfer Vector Core, Iowa City, IA) at a multiplicity of infection of 200 in a volume of 50 μl of complete media for 2 hours, after which, 1 ml of media was added. After 48 or 72 hours, cells were fixed in 4% paraformaldehyde for 20 min to stain for Cre or TUNEL, respectively, or harvested for RNA analysis after 48 hours (*12*, *87*, *88*).

### In vitro dispersed mouse and human islet cell assays

For in vitro studies, mouse and human islets were trypsinized and cultured on glass coverslips in complete media for 24 hours. For the cytokine-treated cell death experiments, the cells were pretreated in serum-free medium with either vehicle, IgG, DMB, or species-compatible OPG, or RANKL (alone or in combination), at specific concentrations for 6 to 8 hours, after which, a mix of species-compatible proinflammatory cytokines, 50 U of IL-1β (mouse, 0.091 ng/ml; human, 0.714 ng/ml), 1000 U of IFN-γ (mouse, 118.62 ng/ml; human, 50 ng/ml), and 1000 U of TNF-α (mouse, 3.7 ng/ml; human, 13.15 ng/ml) (R&D Systems, Minneapolis, MN), or vehicle was added for 16 to 24 hours. For TRAF6 peptide inhibitor studies, mouse islet cell cultures were pretreated for 30 min with either control peptide or TRAF6 inhibitor peptide (Novus Biologicals, Englewood, CO) at 50 μM before addition of the cytokine mix. Cells were fixed in 4% paraformaldehyde (Electron Microscopy Sciences, Hatfield, CA) for 20 min. β cell death was quantified after costaining for TUNEL, insulin, and 4′,6-diamidino-2-phenylindole (DAPI) (*12*, *87*, *88*). The annexin V staining (abcam, Waltham, MA) method was used to assay and quantify mouse β cell death as per the manufacturer’s protocol. Briefly, 16 hours after cytokine addition, dispersed mouse islet cells were stained for 5 min with annexin V and PI, both diluted at 1:100 in the binding buffer. The cells were next washed once with the binding buffer, fixed for 30 min in 2% paraformaldehyde, and then washed again two times with the same buffer. β cell death was quantified after costaining for insulin and DAPI. For immunostaining, mouse islet cell cultures transduced with Adv-Cre or Adv-LacZ for 48 hours were costained for Cre recombinase (1:500, Millipore, Billerica, MA), insulin, and DAPI. Human islet cell cultures treated with vehicle or OPG in the presence or absence of cytokines were costained for p-NF-κB-Ser536 (1:500, abcam, Waltham, MA) or p-STAT1-Ser727 (1:500, Cell Signaling Technology, Danvers, MA), insulin, and DAPI. MFI of p-NF-κB-Ser536 and p-STAT1-Ser727 staining in β cells was quantified in the linear range using the Adobe Photoshop program (*89*). For the human serum–induced cell death studies, human islet cells cultured for 24 hours in control media (containing FCS) or in media in which FCS was substituted for human serum (10% v/v) from T1D or ND subjects in the presence or absence of vehicle (0.1% PBSA), OPG (100 ng/ml), IgG (100 ng/ml), or DMB (100 ng/ml) were fixed and costained for insulin, glucagon (1:500, Abcam, Waltham, MA), TUNEL, and DAPI. For all in vitro studies, samples were analyzed in duplicate in a blinded fashion. An average of 5 and 10 fields, and 893.6 ± 193.7 and 1646.21 ± 124.57 β cells, were quantified per condition for each sample for the in vitro mouse and human islet studies, respectively.

### Static GSIS in mouse and human islets

Isolated mouse islets and human islets were handpicked (15 to 20 IEQs) and cultured in 1.5-ml microcentrifuge tubes with 1 ml of KREBS buffer in low (2.2 mM) glucose for 1 hour. After washing, islets were cultured sequentially with 600 μl of KREBS in low (2.2 mM) glucose for 45 min and then 600 μl of KREBS in high (22.2 mM) glucose for 45 min. Buffers were collected at the end of both low-glucose and high-glucose treatments and frozen. For basal GSIS studies, mouse islets were cultured in the absence or presence of OPG for 45 min. For the stress-induced GSIS studies, the assay was performed on mouse islets cultured in the cytokine mix described above in the presence or absence of IgG or OPG for 16 to 18 hours, and on human islets cultured in serum from ND or T1D subjects as described above in the presence or absence of IgG, OPG, or DMB for 16 to 18 hours. Lastly, islets were extracted with 1 ml of 5% v/v acid-ethanol (to assess total insulin content) and frozen at −20°C. Buffer and acid-ethanol were used for insulin measurements (*87*, *90*).

### INS1 cell assays

The rat insulinoma cell line INS1 shared by H. Komatsu (COH) was cultured in RPMI 1640 media supplemented with 11 mM glucose and 10% FCS. For detection of apoptosis with annexin V/PI staining, INS1 cells were seeded at 300 × 10^3^ cells per well in a six-well plate. After 48 hours, cells were preincubated for 6 hours with vehicle or OPG (1.0 μg/ml) and then treated with vehicle or proinflammatory cytokines (IL-1β, TNF-α, and IFN-γ) at concentrations described above for 16 hours, adding fresh vehicle or OPG. Subsequently, cells were trypsinized with 0.05% trypsin/EDTA (Gibco, CA), washed twice with 1× binding buffer before costaining for annexin V/PI with the TACS annexin V–fluorescein isothiocyanate (FITC) Apoptosis Detection Kit (R&D Systems, MN) according to the manufacturer’s instructions. Flow cytometry data were acquired in a NovoCyte Quantaneon machine (Agilent Technologies, CA) and analyzed in FlowJo software V10 (Tree Star, Ashland, OR). Negative control (unstained cells) as well as annexin V-only– or PI-only–stained cells were used for reading compensation. Live cells were gated for side scatter (SSC) and forward scatter (FSC) size exclusion for analysis. For TRAF6 inhibitor studies, we used two different inhibitors: Compound 6877002, a CD40-TRAF6 chemical inhibitor (Abcam, MA) that inhibits TRAF6 interaction with CD40 and RANK (*35*); and the TRAF6 Inhibitor Peptide Set (Novus Biologicals, CO), specifically disrupting the interaction between RANK and TRAF6 (*36*). For this purpose, INS1 cells were seeded on glass coverslips at a density of 50 × 10^3^ cells per well. After 48 hours, cells were preincubated for 30 min with either vehicle (dimethyl sulfoxide or PBS) or 5 μM of the chemical TRAF6 inhibitor (Abcam, MA) or 30 μM of the control or TRAF6 inhibitor peptide and then treated for 16 hours with vehicle or cytokine mix, as described earlier. Subsequently, cells were fixed with 2% buffered paraformaldehyde and immunostained overnight for cleaved caspase 3 (1:200, Cell Signaling Technologies, MA) and DAPI (*87*, *88*). To visualize intracellular localization of the NF-κB/RELA subunit, INS1 cells seeded on glass coverslips at a density of 50 × 10^3^ cells per well for 48 hours were preincubated for 30 min with either vehicle, control, or TRAF6 inhibitor peptides at 30 μM and then treated with vehicle or cytokines for 30 min, fixed with 2% paraformaldehyde, and immunostained overnight for RelA/NF-κB (1:100, Novus Biologicals, CO) and DAPI. For the INS1 cell death and immunostaining studies, samples were analyzed in duplicate in a blinded fashion, with an average of eight and three fields, and 7678 ± 1208 and 2898 ± 270 cells, quantified per condition for each sample, respectively.

### Protein assays

Insulin, hRANKL, and hOPG from serum or culture media were assayed using ELISA kits for mouse and human insulin (Mercodia Inc., Winston Salem, NC) (*12*, *87*, *91*, *92*), human RANKL (abcam, Waltham, MA), and human OPG (abcam), respectively. IL-1β and IL-6 production in monocytes was determined using ELISA, per the manufacturer’s instructions (BioLegend, San Diego, CA). For Western blot analysis, protein extracts (20 to 40 μg) from human and mouse islets treated with or without cytokines in the presence or absence of IgG, OPG (100 ng/ml), or DMB (100 ng/ml) for 24 hours were analyzed by immunoblotting using antibodies against p-NF-κB-Ser536, p-STAT1-Ser727 (Cell Signaling Technology, Danvers, MA), tubulin (EMD Millipore-Calbiochem, Burlington, MA), and glyceraldehyde-3-phosphate dehydrogenase (Sigma-Aldrich, St. Louis, MO) (table S4). Quantitative densitometry of digitalized blots was performed using the ImageJ program (*12*, *87*, *88*, *90*–*92*).

### mRNA analysis

RNA expression was analyzed by real-time qPCR (ABI 7300; Life Technologies, Carlsbad, CA) using cDNA generated from RNA extracted from mouse or human islets or INS1 cells with RNeasy Mini Kit (Valencia, CA) (*12*, *87*, *89*, *90*). Mouse islet cells from *Rank-floxed* mice transduced with Adv-LacZ or Adv-Cre for 48 hours were analyzed for *actin* and *Rank* expression; human islets (200 IEQs) cultured in media in which FCS was substituted with 10% (v/v) of human sera from T1D or ND control subjects for 24 hours were analyzed for *RANK*, *RANKL*, and *OPG* mRNA levels; and INS1 cells seeded at a density of 500 × 10^3^ cells per well in a six-well plate for 48 hours and treated with either vehicle or mouse cytokines for 8, 16, and 24 hours were analyzed for *Rank* and *cyclophilin*, using species-specific primers (table S5).

### Human serum samples

Blood draws were performed after informed consent was obtained from T1D and ND subjects (table S2) based on the approved Institutional Review Board protocol (no. 15-01137). Serum collected from the blood samples was stored at −80°C. For assaying cytotoxicity of the serum, human islet cells or human islets were cultured in islet media in which FCS was substituted with 10% v/v of either the T1D or ND serum for 24 hours.

### Human PBMC study

Blood samples were collected from healthy donors after obtaining informed consent in accordance with institutional guidelines based on the approved Institutional Review Board protocol (no. 21311). PBMCs were isolated by density gradient centrifugation (Histopaque-1077; Sigma-Aldrich, St. Louis, MO) following the manufacturer’s guidelines. Isolated PBMCs were stimulated with plate-bound anti-CD3 (0.5 μg/ml; OKT3, BioLegend), or purified naïve CD4 T cells were stimulated together with CD28 (1 μg/ml; CD28.2, BioLegend) or CD14^+^ MN. Activated PBMCs and CD4 T cells were collected at 72 or 96 hours after stimulation, respectively, and analyzed by flow cytometry. For proliferation assay, cells were labeled with carboxyfluorescein succinimidyl ester (Invitrogen by Thermo Fisher Scientific, OR) and analyzed by flow cytometry. For intracellular cytokine analysis, cells were stimulated for 4 hours with phorbol 12-myristate 13-acetate (10 ng/ml; Sigma-Aldrich, MA) and ionomycin (1 μg/ml, Thermo Fisher Scientific, MA) in the presence of monensin (1:1000, BioLegend) during the last 2 hours of incubation, and cells were fixed after surface staining and permeabilized with fixation/permeabilization kit (BD Bioscience, CA) and stained with fluorochrome-conjugated antibodies, CD4–phycoerythrin (PE) (RPA-T4), CD8-BV785 (RPA-T8), IFN-γ–allophycocyanin (APC) (4S.B3), TNF-α–BV711 (MAb11), and IL-17–PEcy7 (BL168) (BioLegend). For RANK and RANKL staining, CD4-PE-CF594 (RPA-T4, BD), CD8-BV510 (RPA-T8), CD56-FITC (QA17A16), CD19-Percp-Cy5.5 (HIB19), RANK-PE (9A725, Invitrogen), and RANKL-APC (MIH24, all from BioLegend) were used for surface staining. PBMC CD14^+^ monocytes were isolated using magnetic cell sorting isolation columns (anti-human CD14 kit, positive selection) according to the manufacturer’s protocol (Miltenyi Biotec, Bergisch Gladbach, Germany). CD14^+^ cells were plated at 1 × 10^6^ cells/ml in 96-well plates with 10% human serum albumin (Sigma-Aldrich). For activation, hRANKL (100 ng/ml; ALX-522-012) was added. After 24 hours, medium was collected for analysis of IL-1β and IL-6 cytokines using ELISA (BioLegend).

### Glucose homeostasis

Body weight and blood glucose were measured twice a week. Blood glucose was measured on tail snips using a portable glucometer (Alphatrak, Alameda, CA). IPGTT was performed at 26 to 28 days of treatment in mice fasted for 16 to 18 hours and injected with 2 g of glucose/kg body weight. Plasma insulin was measured on blood drawn at fasting, after 15 min of glucose challenge during IPGTT, and before termination of the study, using an insulin ELISA kit (*12*, *87*, *91*, *92*).

### β cell histomorphometry and immunostaining

Reagents and antibodies used for the in vitro studies are listed in table S4. Pancreata were weighed, fixed in 10% neutral-buffered formalin (Sigma-Aldrich, St Louis, MO), and paraffin embedded. Pancreatic sections were stained for insulin using guinea pig anti-insulin antibody (Dako, Carpenteria, CA) at 1:1000 dilution overnight at 4°C and visualized using the diaminobenzidine peroxidase substrate (Vector Laboratories, Burlingame, CA) and hematoxylin staining. Histomorphometry for β cell mass was performed blinded on four to six insulin-stained pancreatic sections per animal separated by 50 μm each, using the ImageJ program (National Institutes of Health). β cell mass was quantified per animal as the ratio of the insulin-positive to total pancreatic area, multiplied by the pancreas weight, and averaged for all the sections per mouse. β cell proliferation was quantified as percentage of pHH3-insulin to total insulin-positive cells. Pancreatic sections were stained with antibodies against insulin (1:1000) and pHH3 (1:500, Millipore, Billerica, MA), after antigen retrieval with high pressure cooker steam in citrate buffer for 20 min, using an immunofluorescence secondary antibody. β cell death was quantified as percentage of TUNEL-insulin to total insulin-positive cells on pancreatic sections stained with antibodies against insulin and TUNEL (Promega, Indianapolis, IN) (*12*, *87*, *91*, *92*). Insulitis was analyzed on H&E-stained pancreatic sections, by manually scoring the degree of insulitis for every islet in a blinded fashion, and assessing at least 29 ± 2.9 islets per mouse from multiple sections. A previously published formula was used to quantify the degree of insulitis (*93*). The cellular composition of the insulitis was analyzed by three-color IHC staining on Ventana Discovery Ultra IHC Auto stainer (Ventana Medical Systems, Roche Diagnostics, Indianapolis, USA). Briefly, the slides were loaded on the machine, and deparaffinization, rehydration, endogenous peroxidase activity inhibition, and antigen retrieval were performed. The three antigens were then sequentially detected, and heat inactivation was performed to prevent any cross-reactivity between each antigen detection. Following each primary antibody incubation, DISCOVERY anti-Rabbit HQ or NP and anti-HQ-HRP or anti-NP-AP were incubated. The stains were visualized by the DISCOVERY Yellow Kit, Purple Kit, Teal Kit, or Green Kit (Ventana), respectively; counterstained with hematoxylin (Ventana); and coverslipped. IHC-stained slides were digitalized and documented by a NanoZoomer S360 Digital Slide Scanner (Hamamatsu) and viewed by NDP view image viewer software.

### Statistical analysis

Data are expressed as means ± SEM. Statistical significance was considered at *P* < 0.05, *P* < 0.01, *P* < 0.001, and *P* < 0.0001, for single, double, triple, and quadruple symbols, respectively, determined by unpaired two-tailed Student’s *t* test for comparison between two groups; by one-way analysis of variance (ANOVA) with Tukey’s post hoc HSD (http://astatsa.com/) for comparison between more than two groups; by mixed model analysis for repeated measures for the glucose measurements over time; and by the log-rank test for diabetes incidence.
