# Supplementary material for: RANKL/RANK is required for cytokine-induced beta cell death; osteoprotegerin, a RANKL inhibitor, reverses rodent type 1 diabetes
Source: Sci Adv. 2023 Nov 1;9(44):eadf5238. doi: 10.1126/sciadv.adf5238 (PMC10619938; doi:10.1126/sciadv.adf5238)
Supplement: Supplementary file 1 — Figs. S1 to S5 Tables S1 to S5 [file sciadv.adf5238_sm.pdf]

Science Advances
AAAS

Supplementary Materials for

RANKL/RANK is required for cytokine-induced beta cell death; osteoprotegerin, a RANKL inhibitor, reverses rodent type 1 diabetes

Nagesha Guthalu Kondegowda *et al.*

Corresponding author: Rupangi C. Vasavada, rvasavada@coh.org

Sci. Adv. **9**, eadf5238 (2023)
DOI: 10.1126/sciadv.adf5238

This PDF file includes:

Figs. S1 to S5
Tables S1 to S5

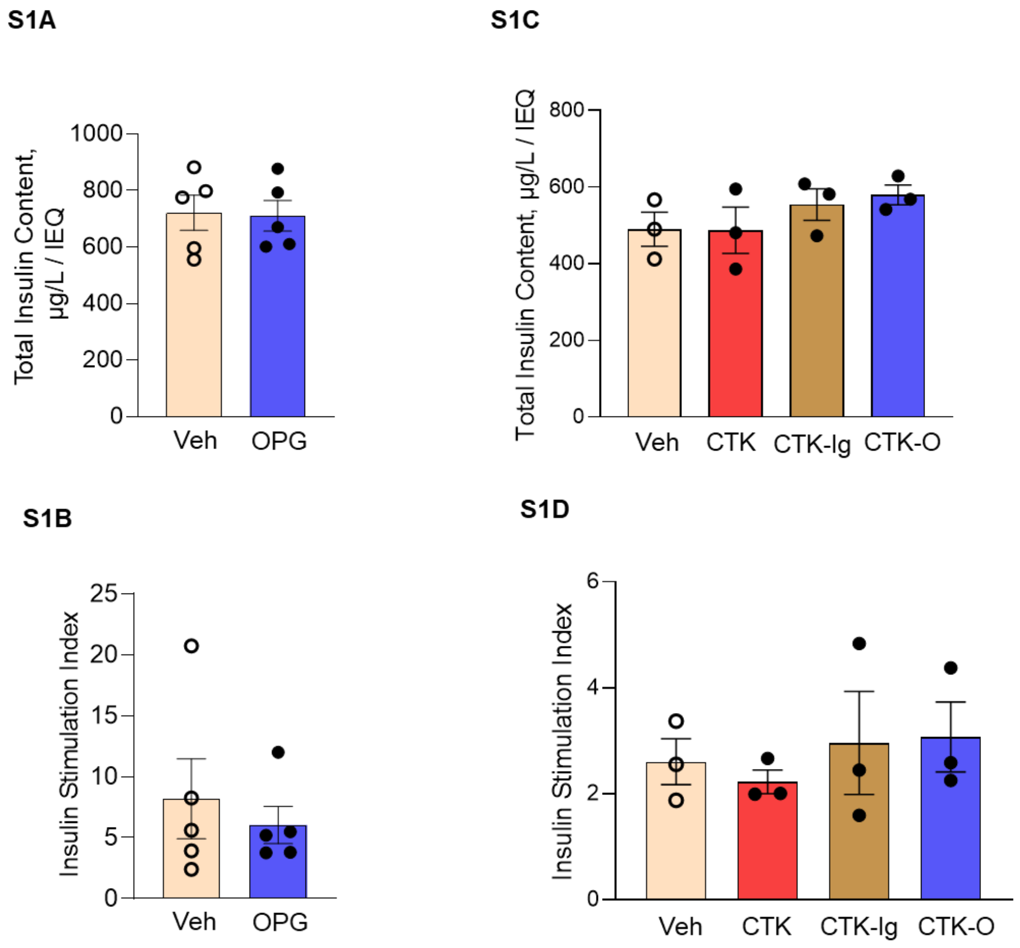

Figure S1. Total insulin content and insulin stimulation index in mouse islets.

(S1A) Total insulin content (µg/L) /islet equivalent (IEQ) and **(S1B)** insulin stimulation index calculated as insulin secreted at 22.2 mM versus 2.2 mM glucose in mouse islets treated with Veh or OPG (100 ng/ml) for 45 min (n=5). **(S1C)** Total insulin content (µg/L)/IEQ and **(S1D)** insulin stimulation index in mouse islets treated with Veh, CTK, CTK+IgG (100 ng/ml) or CTK+OPG (100 ng/ml) for 24h (n=3). Each individual symbol in the graphs represents an independent experiment on different mouse islet preps, averaging duplicate samples for all experiments.

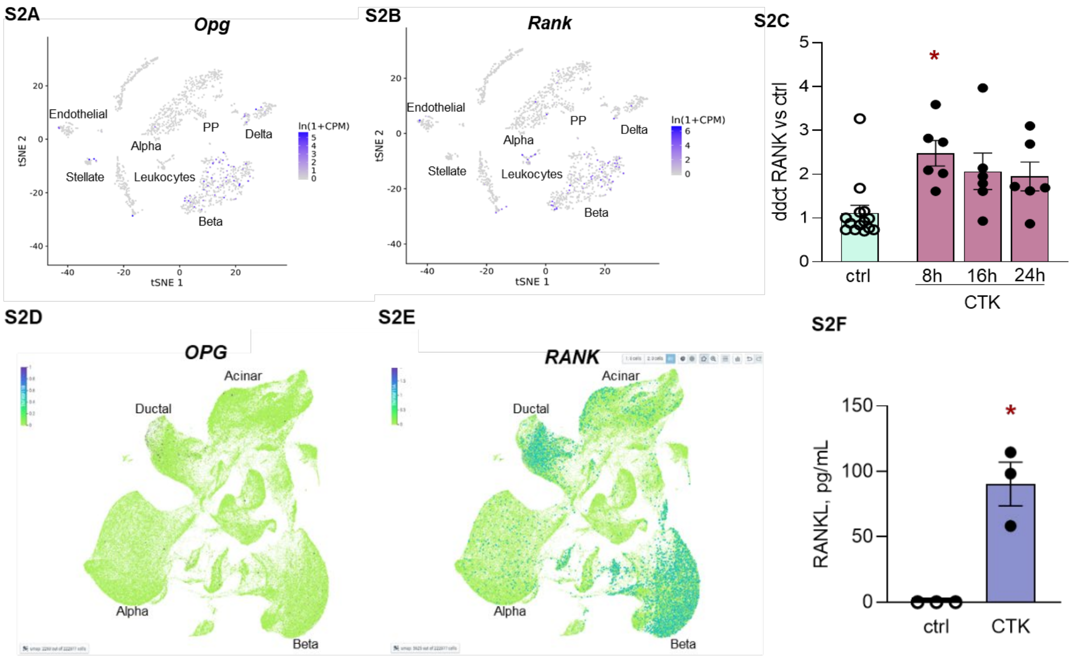

Figure S2. OPG, RANK and RANKL levels in rodent and human pancreas, INS1 cells, and human islets.

t-SNE (t-Distributed Stochastic Neighbor Embedding) representation of single-cell transcriptome analyses based on: https://tabula-muris.ds.czbiohub.org/of **(S2A)** *Opg* and **(S2B)** *Rank* expression in the mouse pancreata, with the different cell type clusters labeled. **(S2C)** qPCR analysis of *Rank* expression versus *Cyclophilin A* as control in INS1 cells treated with vehicle (ctrl) or cytokine mix (CTK) for 8h, 16h or 24h. (n=6) *p<0.05 vs ctrl. t-SNE representation of single-cell transcriptome analyses based on: https://faryabi16.pmacs.upenn.edu/view/T1D_T2D_public.h5ad/ of **(S2D)** *OPG* and **(S2E)** *RANK* expression in the human pancreata, with the various cell type clusters labeled. **(S2F)** Secreted RANKL (pg/ml) from human islets treated with vehicle (ctrl) or CTK for 24h. (n=3) *p<0.05 vs ctrl. Each individual symbol in the graphs represents an independent

experiment on INS1 cells or different human islet preps, averaging duplicate samples for all experiments. All data represent mean ± SEM. Statistical analysis was by t-test (F) and by ANOVA with Tukey's post-hoc analysis (C).

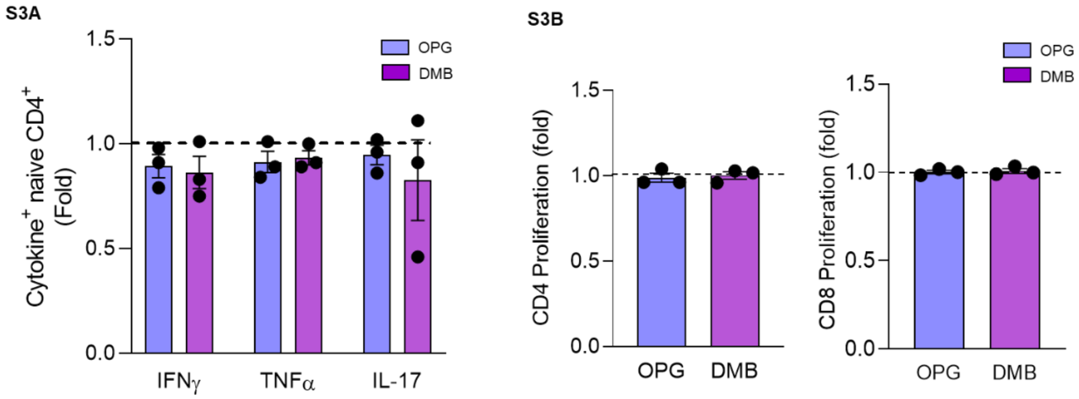

Figure S3. Effects of OPG and DMB on human immune cells.

(S3A) IFNɣ, TNFα, and IL17 cytokine production in CD45RA+ naïve CD4 T cells stimulated with plate bound anti-CD3 (0.5µg/ml) and soluble anti-CD28 (1.0µg/ml) in the absence (untreated) or presence of 500ng/ml each of OPG (blue bar) or DMB (pink bar) for 72h. Intracellular cytokine production is shown on the Y-axis as the ratio (fold) of % cytokine+ cells treated with OPG or DMB versus untreated group. Ratio of 1.0 (dotted line) represents no change in % of cytokine positive cells between OPG or DMB treatment versus no treatment. **(S3B)** Proliferation of CD4 (left panel) and CD8 (right panel) T cells measured by CFSE dilution in PBMCs stimulated with plate bound anti-CD3 (0.5µg/ml) in the absence (untreated) or presence of 500ng/ml each of OPG (blue bar) or DMB (pink bar) for 72h. Y-axis represented as the ratio of values (% of CFSE^{low} T cells) obtained with OPG or DMB versus control untreated group. Each individual symbol in the graphs represents an independent experiment on different human immune cell donors, averaging duplicate samples for all experiments. All data represent mean ± SEM.

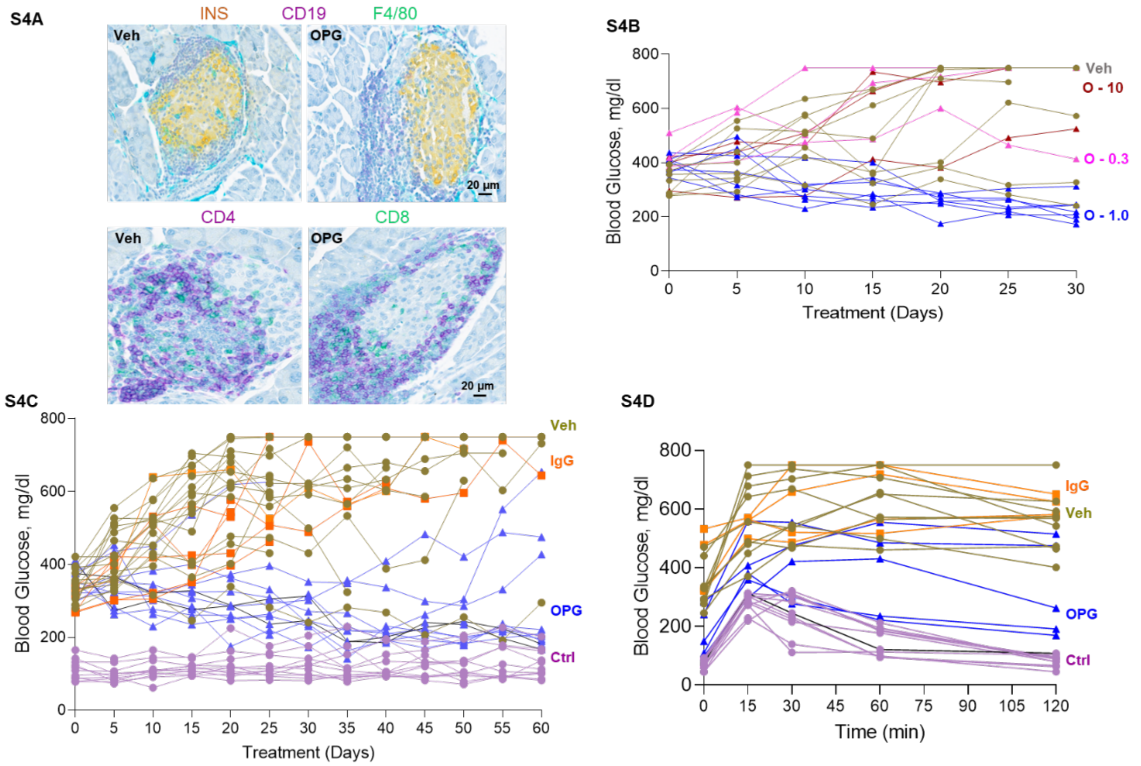

Figure S4. Cellular composition of insulitis and blood glucose of individual NOD mice untreated or treated with vehicle, IgG or OPG.

(S4A) Representative images of pancreatic sections from 11-week-old NOD/Ltj female mice treated daily for five weeks with Veh or OPG at 1.0µg/g (n=3, each) and stained by three-color IHC with antibodies against insulin (brown), CD19 (purple) and F4/80 (teal) (upper panels), to identify β-cells, B-lymphocytes, and macrophages, respectively; or antibodies against FoxP3 (brown), CD4 (purple) and CD8 (teal) (lower panels), to identify T-regulatory cells, CD4+ T-lymphocytes, and CD8+ T-lymphocytes, respectively. Tregs were undetectable in any of the pancreata. Blood glucose in individual NOD/Ltj female mice **(S4B)** treated with Veh (V, olive green, n=8), OPG at 0.3µg/g O-0.3, pink, n=3), 1.0µg/g (O-1.0, blue, n=7), or 10.0µg/g (O-10, brown, n=3) for 30 days, after recent-onset diabetes (defined as blood glucose >250mg/dl for three consecutive days); or **(S4C)** treated

with Veh (olive green, circle, n=16), IgG (1.0μg/g; (orange, square, n=7), or OPG (1.0μg/g; blue, triangle, n=10), for 60 days after recent-onset diabetes, or in untreated control mice that do not develop diabetes (Ctrl, purple, circle, n=11); **(S4D)** at 0, 15, 30, 60 and 120min during an IPGTT in a sub-set (n= 4-11) of the group of mice described above in S4C. Each individual symbol in the graphs represents an individual mouse.

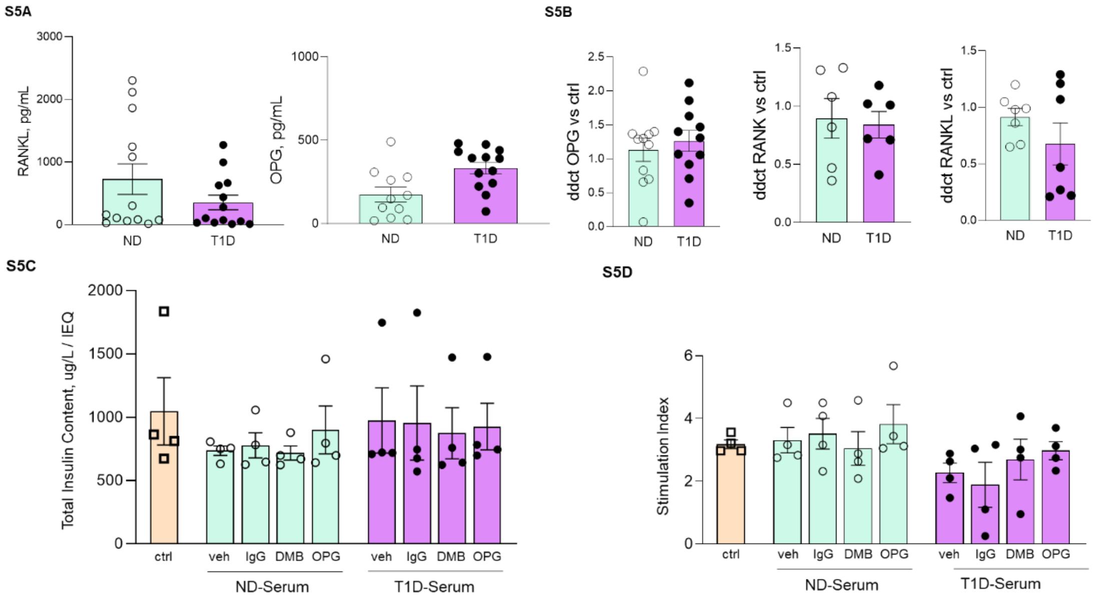

Figure S5. Serum from T1D and ND subjects and their effect on human islets.

(S5A) Levels of RANKL (pg/ml) (left panel) and OPG (pg/ml) (right panel) assessed by ELISA in serum of control nondiabetic (ND) and T1D subjects (n=11-13). **(S5B)** Expression assessed by qPCR of *OPG, RANK* and *RANKL* versus *β-ACTIN* as control in human islets (n=3-4 preps) cultured for 24h in media with FCS replaced by either ND or T1D (n=6-11/group) serum (10% vol/vol). **(S5C)** Total insulin content (µg/L)/IEQ and **(S5D)** insulin stimulation index in human islets (n=2 preps) cultured in regular media containing FCS (ctrl) or in media with FCS substituted with ND or T1D serum (n=4/group) and treated with Veh, or 100ng/ml of IgG, OPG, or DMB for 24h. Each individual symbol in the graphs represents an individual human serum sample tested on human islet preps, averaging duplicate samples for all experiments. All data represent mean ± SEM.

Supplemental Table S1. Summary of glucose homeostasis experiments from Figures 6 and 7

Figure	Experiment	Significance
**Fig 6. A-B**	**Prevent/Delay T1D**	
A	Blood glucose	Yes
B	Diabetes Incidence	No
**Fig 6. C-E**	**Insulitis study**	
C	Blood glucose	No
D-E	Insulitis	Yes
**Fig 6. F-I**	**Reverse T1D; OPG Dose study**	
F	Experimental design	
G	Blood glucose	Yes
H	Blood glucose AUC	Yes
I	Diabetes Incidence	Yes
**Fig 7.**	**Reverse T1D 60 days; IgG control**	
A	Experimental design	
B	Blood glucose	Yes
C	Blood glucose AUC	Yes
D	IPGTT	Yes
E	IPGTT AUC	Yes
F	IPGTT Glucose 0 and 15 min	Yes
G	IPGTT Insulin 0 and 15 min	Yes
H	Insulin D60	Yes
I	Diabetes Incidence	Yes

Supplemental Table S2. Characteristics of non-diabetic control and T1D blood donor subjects

Subject ID	Sex	Ethnicity	Age at Enrollment	Years with Diabetes
**Non-Diabetic Control Subjects**				
7	M	African American, Non-Hispanic	23	NA
8	M	Caucasian, Non-Hispanic	31	NA
9	F	More than one race, Hispanic	45	NA
10	M	Caucasian, Non-Hispanic	57	NA
12	M	Caucasian, Non-Hispanic	23	NA
13	M	Caucasian, Non-Hispanic	29	NA
14	F	Asian	59	NA
27	F	Caucasian, Non-Hispanic	29	NA
28	F	Caucasian, Non-Hispanic	29	NA
31	F	Caucasian, Non-Hispanic	37	NA
36	M	Caucasian, Hispanic	57	NA
56	F	Asian	24	NA
57	M	Caucasian, Non-Hispanic	22	NA
60	M	Caucasian, Non-Hispanic	25	NA
**T1D Subjects**				
1	M	Caucasian, Non-Hispanic	22	1
2	M	African American, Non-Hispanic	24	<1
3	M	Caucasian, Non-Hispanic	23	1
4	M	Caucasian, Non-Hispanic	59	1
5	F	Caucasian, Hispanic	38	<1
6	M	Caucasian, Non-Hispanic	18	<1
11	M	Caucasian, Non-Hispanic	33	3
24	F	Caucasian, Non-Hispanic	36	<1
29	F	Caucasian, Non-Hispanic	68	2
42	F	Caucasian, Non-Hispanic	53	3
46	F	Caucasian, Hispanic	30	2
47	M	Caucasian, Non-Hispanic	28	<1
52	M	Caucasian, Non-Hispanic	47	<1

Supplemental Table S3. **Donor characteristics associated with cadaveric human islets.**

Unique Identifier	Donor Age (Y)	Donor Sex (M/F)	Donor BMI (Kg/m^2)	Donor HbA1c (%)	Source of Islets	Islet Isolation Center
SAMN08774207	21	M	23.9	5.6	IIDP	The Scharp-Lacy Research Institute
SAMN08773854	49	M	40.1	5.4	IIDP	Southern California Islet Resource Center
SAMN08773771	58	F	35.1	4.8	IIDP	Southern California Islet Resource Center
SAMN08930708	56	F	33.1	5.1	IIDP	University of Illinois
SAMN08769818	49	M	27.9	5.6	IIDP	University of Miami
SAMN08930702	64	F	27.4	5.4	IIDP	University of Illinois
SAMN08930703	43	M	36.4	5.5	IIDP	University of Illinois
SAMN08768704	47	F	31.1	NA	IIDP	University of Miami
SAMN08768758	37	F	19.7	NA	IIDP	The Scharp-Lacy Research Institute
SAMN08773766	57	F	20.6	4.9	IIDP	The Scharp-Lacy Research Institute
SAMN08930760	45	M	30.5	5.0	IIDP	University of Illinois
SAMN08769095	47	M	31.0	6.3	IIDP	Southern California Islet Resource Center
SAMN08769086	56	M	40.1	5.8	IIDP	University of Wisconsin
SAMN08769124	15	M	24.6	5.1	IIDP	Southern California Islet Resource Center
SAMN08769122	17	M	39.4	5.1	IIDP	Southern California Islet Resource Center
SAMN08769063	52	F	29.1	5.3	IIDP	Southern California Islet Resource Center
SAMN08768698	60	M	26.6	5.9	IIDP	The Scharp-Lacy Research Institute
SAMN08768977	30	M	31.4	5.3	IIDP	University of Miami
SAMN11982795	54	M	42.8	6.9	IIDP	University of Wisconsin
SAMN12500521	52	M	29.0	5.1	IIDP	University of Wisconsin
SAMN12274306	37	M	25.3	5.2	IIDP	University of Miami
SAMN15314807	27	M	25.3	5.2	IIDP	University of Pennsylvania
SAMN16427178	42	F	31.2	5.5	IIDP	Southern California Islet Resource Center
SAMN15770453	48	F	30.9	5.8	IIDP	University of Wisconsin
SAMN16114998	24	F	45.5	5.2	IIDP	University of Wisconsin
Hu 1158	37	M	24.0	5.5	COH	Southern California Islet Resource Center
Hu1162	31	M	22.0	5.2	COH	Southern California Islet Resource Center
Hu1256	16	M	29.5	5.4	COH	Southern California Islet Resource Center
Hu1257	20	F	36.5	5.8	COH	Southern California Islet Resource Center

Hu1258	47	M	27.8	5.8	COH	Southern California Islet Resource Center
HP-23019-01	69	M	30.4	5.6	Prodo	Prodo Labs
Hu1259	30	F	28.2	5.2	COH	Southern California Islet Resource Center
Hu1260	58	M	23.2	4.6	COH	Southern California Islet Resource Center
HP-23093-01	63	F	29.3	5.2	Prodo	Prodo Labs
Hu1262	39	M	33	5.0	COH	Southern California Islet Resource Center
SAMN31430383	42	F	29.2	7.18	IIDP	Scharp-Lacy Research Institute
Hu1264	47	M	27.5	4.5	COH	Southern California Islet Resource Center

Supplemental Table S4. Reagents

Reagent	Company	Catalog #	Dilution
**Peptides**			
Mouse-OPG-Fc	R&D Systems	459-MO	NA
Human-OPG-Fc	R&D Systems	805-OS-100	NA
Human-OPG-Fc	R&D Systems	6945-OS-025/CF	NA
Denosumab (DMB, Prolia)	Amgen	NA	NA
RANKL peptide	Enzo Lifesciences	ALX-522-012	NA
Human-IgG-Fc	Sigma-Aldrich	AG714	NA
Human-TNF-α	R&D Systems	210-TA-020/CF	13.16 ng/ml
Human-INF-γ	R&D Systems	285-IF-100/CF	50 ng/ml
Human-IL-1β	R&D Systems	201-LB-005/CF	0.72 ng/ml
Mouse-TNF- α	R&D Systems	410-MT	3.704 ng/ml
Mouse-INF-γ	R&D Systems	485-MI	118.62 ng/ml
Mouse-IL-1β	R&D Systems	401-ML	0.091 ng/ml
TRAF6 Inhibitor Peptide Set	Novus Biologicals	NBP2-26506	30-50 μM
**Antibodies**			
p-PHH3-antibody	Millipore Sigma	06-570	1:500
p-NFκB (Ser536)	Cell Signaling Technology	3031	1:1000
p-NFkB (Ser536)	Cell Signaling Technology	3033	1:1000
p-NFκB	abcam	Ab131109	1:500
p-STAT1 (Ser727)	Cell Signaling Technology	9177	1:1000
RelA/NFκB p65	Novus Biologicals	NB100-2176	1:100
Cleaved-Caspase-3	Cell Signaling Technologies	966-1S	1:200
α-tubulin	Millipore Sigma	CP06-100UG	1:2000
tubulin	DSHB	E7	1:1000
Guinea pig anti-Insulin	DAKO	A0564	1:1000
Mouse anti-Glucagon	abcam	Ab10988	1:500
Insulin	abcam	ab96135356	1:1000
Insulin	Cell Signaling Technology	3014 (C27C9)	1:2000
CD4	abcam	ab183685 (EPR19514)	1:200
CD8	Cell Signaling Technology	98941 (D4W2Z)	1:100
CD19	Cell Signaling Technology	90176 (D4V4B)	1:50
FoxP3	abcam	ab215206 (EPR22102-37)	1:100

F4/F80	Cell Signaling Technology	70076 (D2S9R)	1:100
Alex Fluor 488 Goat α-Rabbit	Life Technologies	A11034	1:250
Alex Fluor 594 Goat α-Guinea Pig	Life Technologies	A11076	1:250
Alex Fluor 594 Goat α-Rabbit	Life Technologies	A11037	1:250
Alexa Fluor 488 Goat α-Guinea Pig	Life Technologies	A11073	1:250
Alexa Fluor 488 Goat α-Mouse	Life Technologies	A11029	1:250
HRP Donkey α-Guinea Pig	Jackson ImmunoResearch	706-035-148	1:500
anti-Rabbit HQ DISCOVERY	Ventana	760-4815	1:2000
PE anti-human CD4	Biolegend	300508	1:20
Brilliant Violet 785 anti-human CD8	Biolegend	301046	1:20
APC anti-human IFN-γ	Biolegend	502512	1:20
Brilliant Violet 711 anti-human TNF-α	Biolegend	502940	1:20
PE/Cyanine7 anti-human IL-17A	Biolegend	512315	1:20
PE-CF 594 anti-human CD4	BD Biosciences	562281	1:20
Brilliant Violet 510 anti-human CD8	Biolegend	301048	1:20
PerCP/Cyanine 5.5 anti-human CD19	Biolegend	302230	1:20
FITC anti-human CD56 (NCAM)	Biolegend	318304	1:20
PE-RANK Monoclonal Antibody	Invitrogen	MA1-41015	1:50
APC anti-human CD254 (TRANCE, RANKL)	Biolegend	347508	1:20
Ultra-LEAF Purified anti-human CD3	Biolegend	317326	0.5 μg/ml
Ultra-LEAF Purified anti-human CD28	Biolegend	302934	1 μg/ml
Human TruStain FcX	Biolegend	422302	1:20

**Reagent Kits**			
DeadEnd Fluorimetric TUNEL Kit	Promega	G3250	NA
Annexin-V kit	abcam	Ab14085	1:100
Mouse insulin ELISA kit	Mercodia	10-1249-01	NA
Human insulin ELISA kit	Mercodia	10-1132-01	NA
Human RANKL ELISA kit	abcam	ab213841	NA
Human OPG ELISA kit	abcam	ab189580	NA
TACS Annexin V-FITC Apoptosis Detection Kit	R&D Systems, MN	4830-01-K	NA
DAB Peroxidase Substrate Kit	Victor Labs	SK-4100	NA
ELISA MAX Deluxe Set Human IL-1β	Biolegend	437004	NA
ELISA MAX Deluxe Set Human IL-6	Biolegend	430504	NA
BD Cytofix/Cytoperm	BD Biosciences	51-2090KZ	NA
CellTrace CFSE proliferation kit	Thermo Fisher Scientific	C34554	NA
**Reagents**			
DAPI (4',6-Diamidino-2-Phenylindole)	Life Technologies	D3571	1:200-1000
Collagenase P	Roche	11249002001	NA
Hematoxylin	Sigma-Aldrich	HHS16-500	NA
Eosin	Sigma-Aldrich	RO3040	5%
Hydrogen Peroxide	Sigma-Aldrich	H1009	1:10
Sodium chloride (NaCl 129mM)	Sigma-Aldrich	S7653	NA
Potassium chloride (KCl 4.8 mM)	Sigma-Aldrich	P9333	NA
Calcium chloride dihydrous ($CaCl_2$ $2H_2O$ 2mM)	Sigma-Aldrich	C1016	NA
Sodium bicarbonate ($NaHCO_3$ 24 mM)	Sigma-Aldrich	C5761	NA
Potassium dihydrogen phosphate (KH_2PO_4 1.2mM)	Sigma-Aldrich	10487	NA
Magnesium sulfate heptahydrate (Mg_2SO_4 $7H_2O$ 1.2 mM)	Sigma-Aldrich	1374361	NA

HEPES (6 mM)	Corning	25-060-CI	NA
BSA (0.2%)	Gemini	700-100P	NA
Compound 6877002	Abcam, MA	ab146829	5μM
D-Glucose	Sigma-Aldrich	G7021	NA
PMA	Sigma-Aldrich	P1585-1mg	10 ng/ml
Ionomycin	Thermo Fisher Scientific	J62448	1 μg/ml
Monensin Solution	Biolegend	420701	1:1000
Histopaque-1077	Sigma-Aldrich	10771-100ML	NA

Supplemental Table S5. qPCR Primers

Species	Target	Forward Primer (5' to 3')	Reverse Primer (5' to 3')
**Mouse**	*β-Actin*	GGCCACGGTCTCTTGTTAGA	GGAGACGATAGGGGTGAGGT
	Rank	GCACCCAGGAGAGGCATTAT	CTTCTTCATTCCAGGTGTCCA
**Rat**	*Cyclophilin A*	CACTCAGTCTTGGCAGT	AGCACTGGGGGAGAAAGGATT
	Rank	CCTTGCCTGCATCACAGAC	ATGAGCATCTTGGACGGTGT
**Human**	*β-ACTIN*	CATGTACGTTGCTATCCAGGC	CTCCTTAATGTCACGCACGAT
	RANKL	GTGGCCAACATCCTGCTTAT	AATTCCCTGGGGAAAACTTG
	RANK	TTGAGACCAGGCTGGGTAAC	GGTGCAGCCTCTAACTCCTG
	OPG	CTGGGTTTGCATGCCTTTAT	GGCAACACAGCTCACAAGAA
